# Self-Correcting Maps of Molecular Pathways

**DOI:** 10.1371/journal.pone.0000061

**Published:** 2006-12-20

**Authors:** Andrey Rzhetsky, Tian Zheng, Chani Weinreb

**Affiliations:** 1 Department of Biomedical Informatics, Center for Computational Biology and Bioinformatics and Joint Centers for Systems Biology, Columbia University New York, New York, United States of America; 2 Judith P. Sulzberger MD Columbia Genome Center and Department of Biology, Columbia University New York, New York, United States of America; 3 Department of Statistics, Columbia University New York, New York, United States of America; University of Sheffield, United Kingdom

## Abstract

Reliable and comprehensive maps of molecular pathways are indispensable for guiding complex biomedical experiments. Such maps are typically assembled from myriads of disparate research reports and are replete with inconsistencies due to variations in experimental conditions and/or errors. It is often an intractable task to manually verify internal consistency over a large collection of experimental statements. To automate large-scale reconciliation efforts, we propose a random-arcs-and-nodes model where both nodes (tissue-specific states of biological molecules) and arcs (interactions between them) are represented with random variables. We show how to obtain a non-contradictory model of a molecular network by computing the joint distribution for arc and node variables, and then apply our methodology to a realistic network, generating a set of experimentally testable hypotheses. This network, derived from an automated analysis of over 3,000 full-text research articles, includes genes that have been hypothetically linked to four neurological disorders: Alzheimer's disease, autism, bipolar disorder, and schizophrenia. We estimated that approximately 10% of the published molecular interactions are logically incompatible. Our approach can be directly applied to an array of diverse problems including those encountered in molecular biology, ecology, economics, politics, and sociology.

## Introduction

Scientific innovation often proceeds through a painstaking search for a logically consistent model that best explains a large collection of weakly supported and contradictory facts. We can think of the generation of good models from noisy observations as what John von Neumann called a “synthesis of reliable organisms from unreliable components” [1]. Although scientists are superbly skilled at reasoning over numerous statements of various degree of certainty, this manual reasoning rarely scales up to sets of thousands or millions of statements. This human limitation has become even more obvious during the last decade, due to the emergence of high-throughput techniques that facilitate nearly instant generation of enormous collections of biomedical facts. The main focus of the present study is automatic verification of the consistency of statements about molecular interactions that have been generated by an army of uncoordinated researchers.

To understand the problem at hand, imagine that we need to reconcile data that have been observed by three research laboratories, each of which is unaware of the other's progress. Laboratory 1 ran a series of experiments which strongly suggest that the product of gene *HBP1* is abundant in neurons in the amygdala (a region of the human brain). Laboratory 2 demonstrated that gene *WNT1* is also expressed in the neurons of the amygdala. Laboratory 3 reported experimental evidence that *HBP1*, whenever expressed in a cell, completely inhibits the activity of *WNT1*.

When published in three separate articles and journals, each of these three statements appears reasonable and well-supported; when we combine them, however, we can see clearly that either [1] one of them must be erroneous (for example, the activity of the genes changes over time so that *WNT1* and *HBP1* are never expressed concurrently in the same cell), or [2] we are unaware of an additional fact that can resolve the paradox (such as the existence of a regulator protein that mediates signaling between *HBP1* and *WNT1*).

To make the example slightly more complex (and interesting), imagine that we obtain data from two additional research groups. Laboratory 4's data indicate that the protein *EMX2* is almost certainly expressed in the neurons of the human amygdala; laboratory 5's experimental results show unequivocally that the product of *EMX2* inhibits *WNT1*. Suddenly, we can see that the data that indicate that gene *WNT1* is active in the human amygdala are at odds with the data from the other four laboratories. Thus, laboratory 2's results are the first candidates for reexamination.

Now let us further modify the problem to align it more closely with real world complexity. Imagine that the experimental facts are unequally supported with some showing evidence that is stronger than that of others. Furthermore, instead of having a toy data set that contains just three molecules and only two interactions, we have to deal with facts about presence or absence of hundreds or thousands of molecules that can interact in any number of ways.

## Results and Discussion

### Random arcs-and-nodes model

To address formally the problem that we just outlined, we suggest a *random arcs-and-nodes* graph model—a modified version of a Bayesian network.

The classical Bayesian network formalism was invented to address tasks that resemble that of making an automated medical diagnosis [2]–[7]. A typical Bayesian network had a random variable associated with each node, while the directed arcs of the graph depicted the conditional dependencies between the nodes. For each pair of nodes connected by a directed arc, the node with the outgoing arc was called a *parent* of the node with the incoming arc. The state of each node was assumed to be conditionally dependent on the states of that node's parents, and conditionally independent (given the states of parental nodes) from the remainder of the graph nodes. By design, in these models, the not yet observed states of nodes (unknown disease state that causes the observed symptoms) were of the predominant interest. The arcs—the probabilistic relations between the node variables—were assumed known and immutable [8], [9].

Returning to the problem that we outlined in the introduction, we can see that, when we are dealing with a large collection of statements generated by a diverse set of sources of unequal quality, internal conflicts between the states of numerous arcs and nodes are inescapable. Therefore, it might be useful to allow the arcs themselves to be associated with random variables, and to quantify arc and node-associated uncertainty simultaneously. These new arc-related random variables can represent the strength of the experimental support for individual molecular interactions. We can then update both arc and node distributions, following standard probability calculus, to improve the overall consistency of the model.

Here we suggest a model, a simple generalization of a Bayesian network, where both arcs and nodes represent random variables. As in the classical Bayesian network applied to molecular-biology data, the allowed values for node variables can be defined as *active/present* and *inactive/absent*—which describe the possible states of a molecule in a cell or a tissue. (Alternatively, instead of having only two admissible values per node, we could assume three values: *active, inactive*, and *absent*. For the sake of simplicity, we have chosen to treat the states *inactive* and *absent* as indistinguishable.) Deviating from the classical Bayesian network formalism, we define arc variables, each with allowed values *inhibit, activate*, and *no effect*. The intuition behind this formulation is to provide a mechanism for the arc variables to change their values depending on states of the surrounding nodes, in addition to the traditional probabilistic dependencies between the parent- and child-node variables. (If we assume that the arc variables are conditionally independent of each other and of the node variables, our model reverts to the classical Bayesian network model.) Our goal here is to estimate both the joint and the reconciled marginal distributions over nodes and arcs, given partial prior marginal probabilities on the nodes and arcs and a partial set of conditional probabilities. (To satisfy classic probability calculus, *P*(*A_U_*,_*V*_ = −1)+*P*(*A_U_*,_*V*_ = 0)+*P*(*A_U_*,_*V*_ = 1) = 1, where *inhibit, no effect*, and *activate* are encoded with integers −1, 0 and 1, respectively, and P (*V* = 1)+P (*V* = 0) = 1, where we write *V* = 1 and *V* = 0 for *active/present* and *inactive/absent* values of *V*, respectively.) We can view the reconciled marginal distributions of arcs and nodes in our model as experimentally testable hypotheses.

Random variables associated with arcs can be particularly useful to express general knowledge about molecular events—when it is known that an interaction between two substances is possible, but no precise specification of the condition is given. Node-specific random variables can be useful to express experimental conditions for a specific cell, cell state, tissue, or organ. The initial information about data in our model is expressed as marginal prior probabilities over nodes and arcs. We also define conditional probabilities of nodes given arcs, and of arcs given nodes (see Mathematical Box). We use an analog of the stochastic-integration procedure to compute the joint probability over all random variables. As is common in applications of Bayesian networks to real data, we assume that our molecular-interaction model has no directed cycles.

As will become clear from analysis of examples later in the paper, disparities between prior probabilities and reconciled marginal probabilities emerge when there are substantial conflicts among the prior probabilities for the variables.

### General idea of computation

To make our model applicable to real data, we need a mechanism for estimating a joint distribution of all variables given partial prior and conditional distributions. A good spatial analogy for our proposed computational approach is the problem of inference of a three-dimensional shape (which corresponds to the joint distribution of arc and node variables) of an object, starting with its orthogonal projections (which correspond to the conditional distributions of arcs given nodes and nodes given arcs).

It would be computationally intractable to enumerate explicitly the joint probabilities for all states of all variables in a large random-arcs-and-nodes model due to the enormous size of the state space. However, we can easily define conditional distributions *P*(arcs| nodes are fixed) and *P*(nodes| arcs are fixed) and the prior distributions for all variables. We can then estimate the joint distribution of values for both arcs and nodes by using a Markov chain Monte Carlo technique, which is a computation-efficient version of a stochastic integration [10], [11]. More precisely, we suggest using a Gibbs sampler version of Markov chain Monte Carlo, by sampling values for arcs and nodes from the appropriate conditional distributions, as described in the Mathematical Box and in the Supporting Information.

### Toy and not-so-toy examples

To support our contention that application of our model can lead to intuitive and potentially useful results, we clarify the relevant concepts with three toy examples. From these toy examples it is easy to see that the reconciled marginal distributions correspond to internally consistent pathway graphs. Furthermore, a large change in entropy (loss or gain of information) between the prior and reconciled marginal distributions of random variables is directly attributable to conflicts and agreements among statements in the model. After describing the toy examples we step through a larger, realistic pathway.

**Figure 1:**
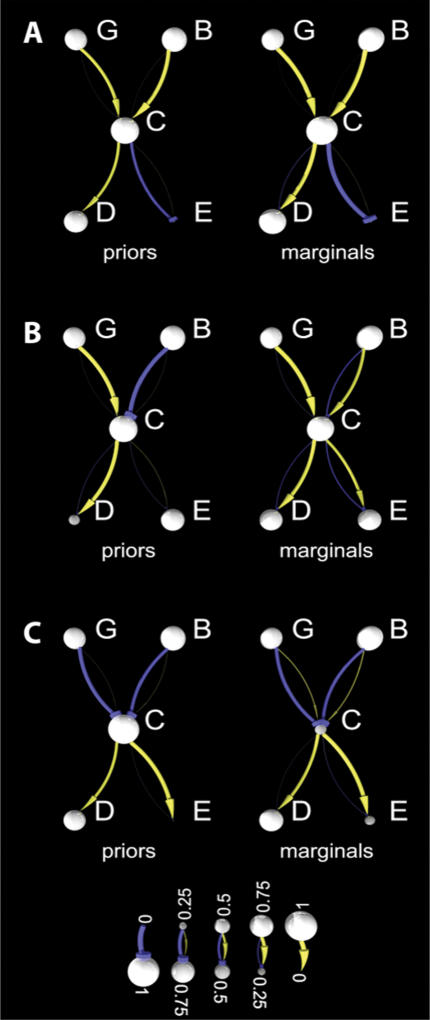
Computation of marginal distributions for all variables (arcs and nodes) of a hypothetical toy graph. A node in the network is a random variable that can have one of two values, *false* or *true* (0 or 1, respectively). Both the brightness and the size of a node represent the strength of the probability that the corresponding molecule is *present/active* in the tissue or cell of interest, *P*(*Vi* = 1). A higher probability is depicted with a lighter color and larger ball radius (see key to the node color and size); when the *P*(*Vi* = 1) drops to 0, the node disappears from the figure (the ball radius drops to zero). Each arc is a random variable with three possible different values: *inhibit, activate*, and *no effect* (*−1*, 1, and 0, respectively). Complete confidence that an arc *A_V,U_* represents an inhibiting function (*P*(*A_V,U_* = −1) = 1) would be drawn as a thick bright-blue edge with a disk at the end (the leftmost edge in the key to the figure). If both probabilities (*P*(*A_V,U_* = −1) and *P*(*A_V,U_* = 1)) drop to zero (indicating that *P*(*A_V,U_* = 0) = 1), then the edge vanishes from the figure, indicating the *no effect* value. (A) An internally consistent set of prior probabilities. The resulting marginal distributions are either unchanged (on the input nodes G and B and on the sink node E) or have a decreased entropy (on all arcs and on nodes C and D), in contrast to the prior probabilities. (B) An example with inconsistent prior probabilities. The marginal distribution for arc *A_BC_* is reversed with respect to the prior. (C) Another example of conflicting prior probabilities. Here, node C changed its distribution significantly.

For our toy example we have chosen an X-shaped directed graph shown in Figure 1. We look at three different prior variable distributions for the same-topology. Figure 1 (A) has logically consistent prior distributions over the variables. The most likely states of nodes G, B, and C are *active/present*; consistent with that, G and B both, most probably, activate C. Similarly, node C (most probably) inhibits node E and activates node D, a situation consistent with the probable states of nodes D and E, respectively. The reconciled marginal distributions for the same variables (Figure 1 (A), marginals) are visually similar to the corresponding prior distributions. However, the reconciled marginal distribution on average became more informative: the overall entropy of the reconciled marginal distributions drops by 0.45 bits for the node variables and by 2.14 bits for the arc variables, in comparison to the prior distribution. (The Shannon entropy of a random variable with just two states, 0 and 1, is defined as -*p*_0_ log_2_ *p*_0_ -*p*_1_ log_2_ *p*_1_, where *p*_0_ and *p*_1_ are the probabilities that we will find the variable in state 0 or 1, respectively. A similar expression with three terms in the sum defines the entropy of a three-state random variable. The Shannon information is defined as a difference between two values of entropy for the same system; information is gained when entropy decreases and is lost when entropy grows.) In other words, if we start with a set of logically consistent prior distributions over variables in a graph, we can gain information by computing the joint distribution over all variables, because consistent parts of the random graph reinforce one another and make the reconciled marginal distribution sharper (more informative).

The inconsistent prior distributions for the same variables (Figure 1 (B) and (C)) lead to quite different properties of the reconciled distributions. In the graph shown in Figure 1 (B), node B is active and is believed to inhibit node C, yet C is believed to be active. In addition, node C is believed to activate node D, yet node D is most likely inhibited/absent. The corresponding reconciled marginal distributions for arcs and nodes are no longer inconsistent: node D becomes activated, while arc _ABC_ changes its most likely value from *inhibit* to *activate*. However, this improvement in consistency is achieved at a price: loss of certainty in the reconciled marginal distributions. That is, the entropy for the reconciled distributions increases by 1.41 bits for nodes and by 0.32 bits for arcs. The example in Figure 1 (C) has an apparent conflict between the states of arcs *A*_GC_ and *A*_BC_ (both arcs are, most likely, in the state *inhibit*) and the *active/present* states of nodes G, B and C. In addition, node E is originally believed to be activated by node C, but its most likely state is *inactive*. As with the previous examples, the reconciled marginal distributions are free of the inconsistencies observed in the prior distribution, but at the expense of an increase in the entropy (loss of information, by 0.125 bits for nodes and 0.53 bits for arcs). A larger, realistic pathway graph can have both consistent and contradictory parts.

**Figure 2:**
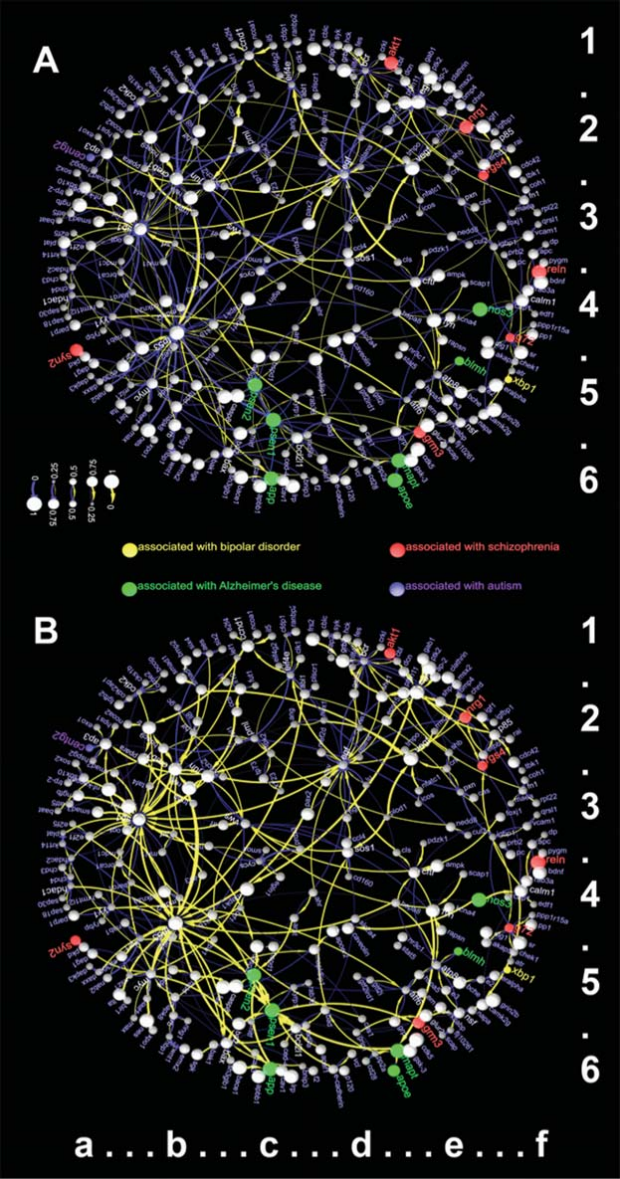
Distributions for all arc and node variables in a large human molecular network. (A) Prior distributions for arcs and nodes computed by automated analysis of thousands of research articles. (B) Reconciled marginal distributions for all variables in the graph: The graph has changed to improve the consistency of individual pieces of information, some of which were conflicting in the graph A. Green, blue, yellow, and red nodes correspond to genes that were previously reported as associated with Alzheimer's disease, autism, bipolar disorder, and schizophrenia, respectively. The nodes that we mentioned in the text have the following coordinates: *WNT1 (6b), HBP1 (6b), EMX2 (6b), SRF (3b), SP1 (3b), TP53 (4b), PSEN1 (5c)*.

**Figure 3:**
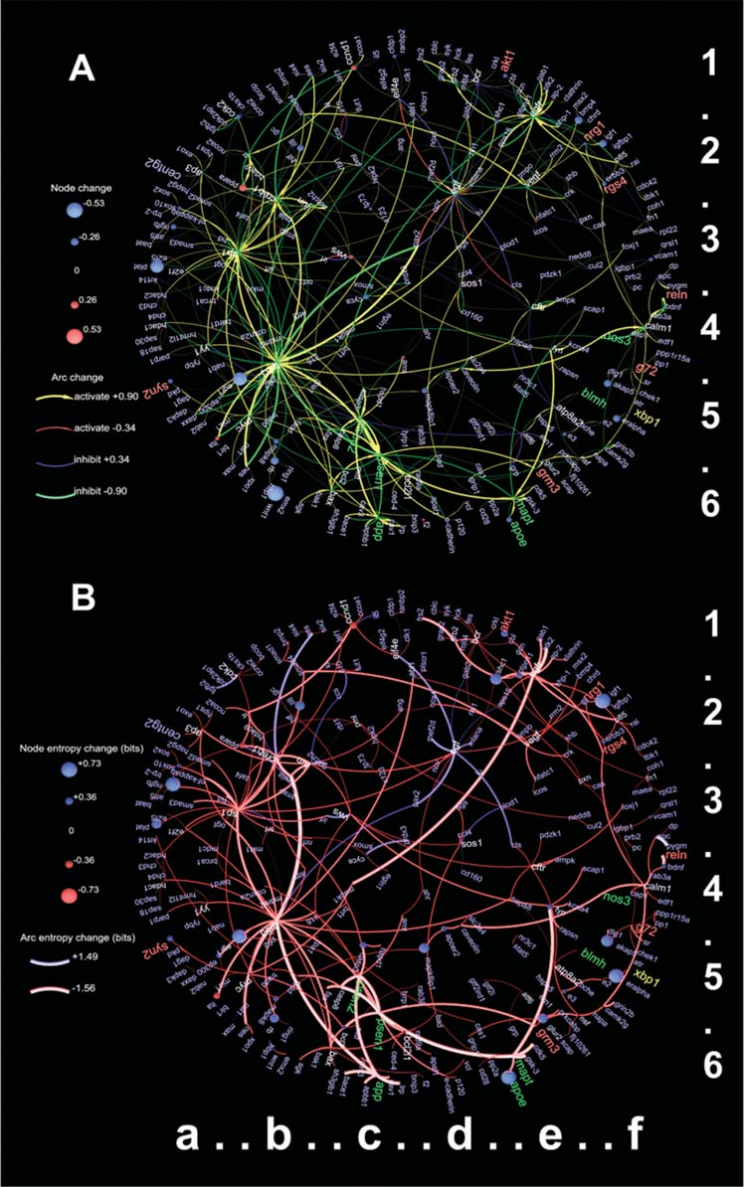
Figure 3. Difference and entropy-change graphs for networks shown in Figure 2. (A) The absolute difference between the reconciled (Figure 2 B) and the prior (Figure 2 A) distributions. For the *activate* arc value, an increase in probability is shown in yellow; a decrease is shown in red. Similarly, for the *inhibit* arc value, an increase in probability is shown in blue; a decrease is shown in green. For graph nodes, positive changes (increases) in the probability of observing the node in the *active/present* state are shown in red; negative changes (decreases) are shown in blue. (B) Differences in the Shannon entropy (bits) for arc and node variables between the reconciled and prior marginal distributions. Red variables lost their entropy (gained information), whereas blue ones increased their entropy (lost information), after computation of reconciled distributions. The nodes that we mentioned in the text have the following coordinates: *WNT1 (6b), HBP1 (6b), EMX2 (6b), SRF (3b), SP1 (3b), TP53 (4b), PSEN1 (5c)*.

To get a large, experimentally grounded data set, we used data from a large-scale text-mining project [12], [13] that provided access to experimental results described in hundreds of thousands of published research articles. These data closely match the imaginary situation described earlier, where researchers at numerous laboratories ran experiments unaware of each other's results [14]. We decided to compile and analyze a set of human molecular interactions among genes that are suspected to harbor genetic polymorphisms predisposing to one of four major neurological disorders: autism, Alzheimer's disease, bipolar disorder, and schizophrenia. We present here analysis of 3, 161 full-text articles (we used 6, 724 unique sentences from these articles to extract molecular interactions) from 64 major scientific journals (see Supporting Information for detailed information on sources of data). The molecular network that we analyzed with our method was devoid of directed cycles; to generate a loopless graph, in each directed cycle of the original literature-derived network model, we removed the weakest (least supported) arc, striving to minimize the overall number of deleted arcs. To collect information on the brain-specific expression of genes in our molecular network, we examined 910, 221 journal abstracts that specifically referred to brain tissues; 14, 780 of these abstracts mentioned genes that we selected for our example (see Supporting Information for more detail). The result of this analysis was a molecular network that comprised 288 nodes and 353 arcs; each arc was represented by multiple statements and types of interactions from the literature. (We could have analyzed a much larger network, but the results would not have been amenable to compact representation easily accessible to a reader; nonetheless, our current pathway model, presented in Figures 2 and 3, is much larger than a typical pathway described in a comprehensive review article.)

In this large molecular network, we defined the prior distributions for the node variables using published statements about tissue-specific expression of individual genes. We computed the prior distributions for the arcs using the individual relationships between molecules extracted from the literature combined with the estimated confidence in the quality of the extraction of the individual relations (see Mathematical Box and Supporting Information for details).

We visualized the prior and reconciled marginal distributions side-by-side in Figure 2 to facilitate their comparison, and showed the absolute difference between them in Figure 3 (A). Additionally, we computed the change in entropy between the prior and reconciled distributions for each individual random variable (Figure 3 (B)). The difference in entropy highlights the consistent and inconsistent parts of the graph: the blue-spectrum nodes and arcs increased their entropy (lost information), while the red-spectrum variables lost entropy (gained information). The blue-spectrum variables are the best candidates for further experimental corroboration or refutation.

We begin the analysis of our realistic pathway example by observing that the hypothetical example which we posited in the introduction exists in the real-life example. According to published statements, gene *WNT1* is inhibited by both *HBP1* and *EMX2* [15], [16]. Therefore, the pathway, as represented by the set of prior distributions over variable values, is inconsistent.

One of the arcs that decreased its activate (associated reconciled marginal probability) is the one connecting *SRF* to *SP1* (see Figures 2 and 3). It also shows a loss of information (it has a blue connecting line in 3 (B)). If we trace the arc support back to the source papers, we find that this particular arc is supported by a single sentence that formulates a hypothesis: “The combination of increased *JNK* activity and up-regulation of *c-JUN* and related proteins may activate gene transcription via interactions between *c-JUN, SRF*, and the trans-activation domain of *SP1*.” (see [17]).

Some of our arc reconciled marginal distributions appear to conflict with the published data. One of the prominent examples of this kind in our figure is the interaction between *TP53* (a notorious transcription factor participating in a number of cancer- and cell-death related pathways) and *PSEN1* (human gene that is believed to harbor polymorphisms predisposing the bearer to Alzheimer's disease). Our prior distribution for this arc indicated that *TP53* inhibits *PSEN1* (*e.g.*, see [18]–[20]). Yet our prior distributions for the nodes *TP53* and *PSEN1* were strongly biased towards *active/present* state. Furthermore, according to our compiled graph, both *TP53* and *PSEN1* are activated by a number of other genes (*TP53* is activated by *EGR1* and *TRRAP*, while *PSEN1* is activated by *e-CADHERIN*, and *BCL-2*), further supporting the hypothesis that both genes are active. As a result, the reconciled distribution for the arc between *TP53* and *PSEN1* has a larger probability for *activate* than for *inhibit* (see Figure 2). This apparent inconsistency can be explained and resolved in a number of ways. The interaction between *TP53* and *PSEN1* may be in reality mediated by a third gene that is inactive in the neurons. An alternative explanation is that *TP53* and *PSEN1* are indeed active in the same neuronal cells, but not at the same time. This can be tested by looking at experimental time series reflecting changes in states of genes proteins and other molecules in a cell.

Our computational approach identified inconsistencies in states of approximately 10% of arcs and 8% of nodes within the realistic pathway graph (see Figures 2 and 3). We hypothesize that these estimates reflect the overall level of inconsistency among the published statements about molecular interactions.

Figures 2 and 3 point to dozens of experimentally testable hypotheses that, we hope, the reader would be tempted to examine. Using the proposed methodology and currently accessible computational resources, we can scale the computation up to apply to thousands or even millions of statements, potentially, to the complete set of human molecular interactions.

### Relation to other computational approaches

Recent probabilistic approaches, successfully applied to the analysis of molecular pathways, were built on either treating physical molecular interactions (arcs) as discrete model parameters (e.g., see [21], [22]) or describing expression levels of genes (nodes) as random variables related to one another according to immutable non-contradictory conditional distributions [8], [23] learned from experimental data [3], [8], [23]. The model that is the closest to our model [21] used both discrete variables for nodes (gene expression levels after gene knockout experiments) and discrete parameters for arcs to infer molecular pathways from experimental data. The approach that we propose here is different both in the goal (improving internal consistency of large graphs by refuting or strengthening individual facts) and in the methodology, which describes both nodes (states of molecules) and arcs (dependencies between nodes) as random variables defined within a unified probabilistic model. In addition, we use a stochastic integration technique (a Gibbs sampler) to estimate the joint distribution for all variables in our model. Our model belongs to a large family of factor-graph models [24] and, to the best of our knowledge, has not been suggested before our current study.

### Extensions and conclusion

A natural next step is to use our model to integrate results from large-scale wet-laboratory experiments with text-mining analyses statements. We hope to expand our methodology by incorporating the ability to handle directed cycles which are critically important in biological pathways. We can significantly improve (while making it also more complicated) the model for assigning the prior probabilities for nodes and arcs. For example, we can use a probabilistic mode of scientific publication process [13] to take into account the type and amount of experimental support behind the published statements. A more long-term goal is to assemble and cross-validate a reliable and comprehensive map of human interactions, to enable diagnosis and treatment of complex human disorders [25]. Since molecular networks of distinct species interact with each other, as is clear in the case of the pathogens and various allergy-inducing agents in humans, it is not unimaginable to attempt computing a reconciled model of the whole integrated current knowledge about molecular interactions [14]. Finally, we can imagine a futuristic environment where new molecular-interaction hypotheses are automatically tested for consistency against the set of currently available facts.

Once a proper mapping of arc and node variables is defined, our model is immediately applicable to a diverse set of problems outside of molecular biology. For example, in ecology the node variables can represent presence or absence of a species in a geographic location, while arcs can represent predator-prey, host-parasite, mutualism, or synergism inter-species relations [26]. In sociology the nodes can represent individuals present or absent in different groups while arcs can represent dependencies or associations between people [27]. In political sciences the nodes can represent countries and their interactions in the context of local conflicts and economic competition [28]. In economics, the graph nodes map to companies which may be either active or inactive in various markets, and the arcs depict collaboration, competition, or dependence between the various businesses. The common feature unifying all these disparate networks is that each of them has to be assembled from a rapidly growing avalanche of conflicting observation of unequal quality that need to be reconciled at a large scale.

## Methods

### Mathematical Box

#### Node prior-probability values

Imagine that our text-mining machinery can identify in research papers statements of the form *“gene A is active in tissue B”* (or *“small molecule A is present in cell B”)*. Furthermore, assume that we treat all such statements as equally reliable, and that we have exactly *n_AB_* of them, with each statement appearing in a separate article. Then, we can define the prior probability for a specific gene or molecule *A* to be present (active) in tissue *B*:
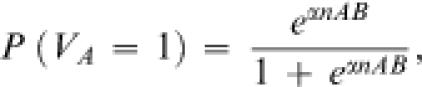
1where *α* is a positive-valued parameter that we introduce to scale down the counts *n_AB_* so that a near-absolute prior certainty is achieved only at very large values of *n_AB_*. Following the same logic, we can define *negative* counts for *n_AB_* to indicate negative statements (*“gene A is not active in tissue B”*). In the absence of data (*n_AB_* = 0), we would have an uninformative prior: *P*(*V_A_* = 1) = *P*(*V_A_* = 0) = 1/2. We would obtain a prior-probability value greater than 1/2 if *n_AB_* is positive, and less than 1/2 if *n_AB_* is negative.

In practice, we can, approximate counts* n_AB_* with the number of co-occurrences (say, within the same sentence) of the terms *A* and *B* in the research literature.

#### Arc prior-probability values

To compute prior-probability distributions for arcs, we start with an estimate of the probability that we correctly extracted the statement. Assuming that we extracted the statement *substance i activates substance j* from *N* distinct sentences, and knowing the probability that the *k* ^th^ instance was extracted correctly, we can define the prior confidence in the corresponding arc:
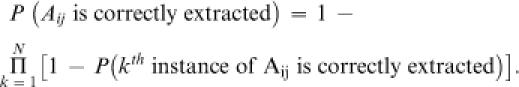
2


This equation follows the simple logic that, for an arc supported by multiple independent statements to be incorrectly extracted, all of the independently extracted instances of supporting statements instances of the fact must be incorrectly extracted.

In the absence of specific knowledge about inhibiting or activating effect of arcs (such as *phosphorylate*), the prior distribution was distributed uniformly over inhibiting and activating values of the arc. Whenever specific statements supporting an inhibiting or activating value of a particular arc become available, we compute the prior distribution for the arc by first using Equation 2 separately, for all activating, inhibiting, and sign-less labels of arcs (*p_a_*, *p_i_* and *p_p_*, respectively), and then assigning probabilities
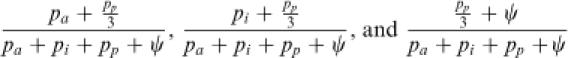
to the prior distribution over *activate, inhibit*, and *no effect* values for the arc, respectively. (Parameter ψ is set to a small positive value that ensures that the prior-probability distribution for an arc has correct properties even in the absence of data.)

We can further improve the prior distribution estimated for an arc by taking into account the estimated probability that the statement is true given its publication patter (we can obtain such an estimate, for example, by using the model of the chain of collective reasoning [29]).

#### Arc update

The conditional probabilities for arc *A_ij_* given its adjacent nodes, *V_i_* and *V_j_*, are defined in the following way:
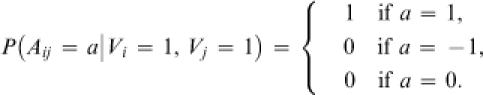
3

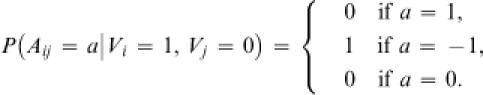
4

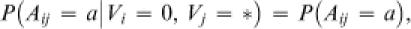
5where * is a wildcard symbol that represents both 1 and 0.

#### Node update

The conditional probability for a node given assigned values of parental nodes and arcs is defined as follows:
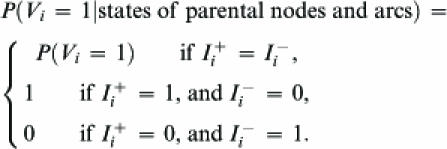
6where *I* ^+^ *_i_* = 1 if at least one of the parents of the *_i_* ^th^ node is in an active state and the arc leading from the parent to child node *i* is in the state *activate* (otherwise, *I* ^+^ *_i_* = 0); similarly, *I* ^−^ *_i_* = 1 if one or more of the parents of the *_i_* ^th^ node is in an active state and is connected to the *i* ^th^ node by an inhibiting arc. Finally,
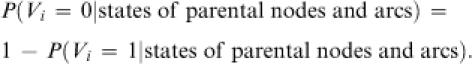
7


We developed and tested a number of alternative models for updating nodes given arcs and arcs given nodes (see Supporting Information). These alternative models are more parameter-rich but less restrictive in assumptions about resolution of apparent conflicts between prior distributions of node and arc variables than the simplest model described here.

#### The Gibbs sampler

The stochastic update of node and arc variable values is performed in the following way.

**The zero^th^ step:** With probability 1/2, we start with updating arcs values (or node values). If we decided to start with arcs, we proceed as follows:

**The first step:** We sample the value for each arc, *A_ij_* from that arc's prior distribution, *P*(*A_ij_ = a_ij_*), where *a_ij_* = 1, 0, or −1. **The second step:** Having assigned values to the arcs, we update the values of nodes, starting with input nodes in the graph. Values for the input nodes (also called *external* nodes, or *parentless* nodes) are sampled from the prior distributions for these nodes. The update proceeds down to the sink (*childless*) nodes, sampling the value for each child node from the conditional distribution P (*Vi* = *vi*|{*Vj* = *vj*, *A_ij_* = *a_ij_*}*_Vj_*_∈ *par*(*Vi*))_. (Notation “*V_j_* ∈ *par*(*V_i_)”* stands for “*V_j_* belongs to the set of parents of node *V_i_*.” Node *V_j_* is called a parent of node *V_i_* if there is a directed arc, *A_ji_*, from node *V_j_* to node *V_i_*. ) **The third step:** Having assigned values to the nodes, we update values of arcs, sampling the value for each arc from the following conditional distribution: *P*(*A_ij_* = *a_ij_*|*V_i_ = v_i_*, *V_j_ = v_j_*). Given the states of the flanking nodes, arcs are independent with regard to one another and thus can be sampled individually in any order. **The fourth step:** We run steps 2 and 3 for a large, predefined number of times, recording values of arcs and nodes after each complete update of those values.

If we decide to start with nodes at the step 0, then we proceed as follows.

**The first step:** We generate values for nodes from the prior distribution for each node, *P*(*Vi = vj*). **The second step:** We generate values for arcs; the value for arc _Aij_ is sampled from the conditional distribution P (*A_ij_ = a_ij_*|*V_i_ = vi*, *Vj = vj*). **The third step:** Having assigned values to the arcs, we update the values of nodes, starting with input nodes in the graph and proceeding down to the sink nodes, sampling the value for each child node from the conditional distribution *P*(*Vi* = *vi*|{*Vj* = *vj*, *A_ij_* = *a_ij_*}*_Vj_*_∈ *par*(*Vi*)_). **The fourth step:** We run steps 2 and 3 for a large, pre-defined number of times, recording values of arcs and nodes after each complete update of these values.

We estimate the joint distribution of values for arcs and nodes by running the Gibbs sampler numerous times, each time randomly deciding whether to start with arc or node update. We obtain the distribution estimate by computing the frequency of states (values of arcs and nodes) visited by the Gibbs sampler in a large number of independent runs. Many independent runs are required because prior belief conflicts make the joint distribution multimodal: Each mode corresponds to one of the ways of resolving conflicts.

We evaluated the convergence of the Gibbs sampler by direct comparison to the exact distributions computed for a Bayesian network with estimates provided by the Gibbs sampler (see Supporting Information). It appears that the stochastic procedure (Gibbs sampler) converges fairly quickly (tens of thousands of independent chain starts and hundreds to thousands of iteration within each such run).

## Supporting Information

Supplement S1 Additional information on mathematics of the method
(1.93 MB PDF)

Supplement S2 Additional information about dataset used
(0.61 MB PDF)
