# Supplementary material for: Self-Correcting Maps of Molecular Pathways
Source: PLoS One. 2006 Dec 20;1(1):e61. doi: 10.1371/journal.pone.0000061 (PMC1762305; doi:10.1371/journal.pone.0000061)
Supplement: Supplement S1 — Additional information on mathematics of the method (1.93 MB PDF) [file pone.0000061.s001.pdf]

Supplement

March 30, 2006

Contents

1 Supplement A. Arcs update **4**
- 1.1 Arcs update version A . . . 4
- 1.2 Arcs update version B . . . 4
- 1.3 Arcs update version C . . . 5
- 1.4 Arcs update version D . . . 5
- 1.5 Arcs update: Version E . . . 6
- 1.6 Multiple arcs connected to the same node . . . 7

2 Supplement B: updating nodes given fixed arcs **8**
- 2.1 Node update version A . . . 8
- 2.2 Node update version B . . . 9
- 2.3 Node update version C . . . 9

3 Supplement C: enumeration examples **10**
- 3.1 Independent arcs . . . 10
- 3.2 Linear example . . . 10
- 3.3 X-shaped example . . . 11
  - 3.3.1 Independent arcs . . . 12
  - 3.3.2 Updating both nodes and arcs . . . 14

4 Supplement D. Graph support **15**
- 4.1 List of Nodes . . . 15
- 4.2 List of Arcs . . . 15
- 4.3 List of Arc Types . . . 15
- 4.4 List of Brain Tissue Names . . . 15

List of Figures

1 Update of both arcs and nodes of a hypothetical five-node and four-arc network (node update version A, arc update from priors). 13
2 Update of both arcs and nodes of a hypothetical five-node and four-arc network (node update version A, arc update version C—detailed numerical information corresponding to this figure is shown in Tables 4 and 5). 16
3 Update of both arcs and nodes of a hypothetical five-node and four-arc network (node update version A, arc update version D (equivalent to version E with $\beta = 1$)—detailed numerical information corresponding to this figure is shown in Tables 6 and 7). 17
4 Update of both arcs and nodes of a hypothetical five-node and four-arc network (node update version B, arc update version B—detailed numerical information corresponding to this figure is shown in Tables 8 and 9). 18
5 Update of both arcs and nodes of a hypothetical five-node and four-arc network (node update version B, arc update version C—detailed numerical information corresponding to this figure is shown in Tables 10 and 11). 20
6 Update of both arcs and nodes of a hypothetical five-node and four-arc network (node update version B, arc update version E (with $\beta = 0.2$)—detailed numerical information corresponding to this figure is shown in Tables 12 and 13). 21

List of Tables

1 Marginals for the nodes exact and estimated with the Gibbs sampler: node update version A. (Gibbs sampler: $50,000$ different starting points, 100 iterations for each chain.) 12

2 Marginals for the nodes—comparison of the exact computation with the Gibbs sampler: node update version B.(Gibbs sampler: $50,000$ different starting points, 100 iterations for each chain.) 13

3 Versions of update depicted in the supplement. 14

4 Marginals for the nodes estimated with the Gibbs sampler: node update model A, arc update model C. (Gibbs sampler: $10,000$ different starting points, 100 iterations for each chain.) 14

5 Marginals for the arcs estimated with the Gibbs sampler: node update model A, arc update model C. (Gibbs sampler: $10,000$ different starting points, 100 iterations for each chain.) 15

6 Marginals for the nodes estimated with the Gibbs sampler: node update model A, arc update model D. (Gibbs sampler: $10,000$ different starting points, 100 iterations for each chain.) 15

7 Marginals for the arcs estimated with the Gibbs sampler: node update model A, arc update model D. (Gibbs sampler: $10,000$ different starting points, 100 iterations for each chain.) 16

8 Marginals for the nodes estimated with the Gibbs sampler: node update model B, arc update model B.(Gibbs sampler: $50,000$ different starting points, 100 iterations for each chain.) 17

9 Marginals for the arcs estimated with the Gibbs sampler: node update model B, arc update model B. (Gibbs sampler: $50,000$ different starting points, 100 iterations for each chain.) 18

10 Marginals for the nodes estimated with the Gibbs sampler: node update model B, arc update model C. (Gibbs sampler: $10,000$ different starting points, 100 iterations for each chain.) 19

11 Marginals for the arcs estimated with the Gibbs sampler: node update model B, arc update model C. (Gibbs sampler: $10,000$ different starting points, 100 iterations for each chain.) 19

12 Marginals for the nodes estimated with the Gibbs sampler: node update model B, arc update model E (with $\beta = 0.2$). (Gibbs sampler: $10,000$ different starting points, 100 iterations for each chain.) 19

13 Marginals for the arcs estimated with the Gibbs sampler: node update model B, arc update model E (with $\beta = 0.2$), (Gibbs sampler: $10,000$ different starting points, 100 iterations for each chain.) 20

1 Supplement A. Arcs update

In this section, we consider different schemes of conditional probabilities for updating one arc, A_{ij} given its adjacent nodes, V_i and V_j.

1.1 Arcs update version A

$$P(A_{ij} = a|V_i = 1, V_j = 1) = \begin{cases} 1 - P_0(A_{ij} = 0) & \text{if } a = 1, \\ 0 & \text{if } a = -1, \\ P_0(A_{ij} = 0) & \text{if } a = 0. \end{cases} \tag{1}$$

$$P(A_{ij} = a|V_i = 1, V_j = 0) = \begin{cases} 0 & \text{if } a = 1, \\ 1 - P_0(A_{ij} = 0) & \text{if } a = -1, \\ P_0(A_{ij} = 0) & \text{if } a = 0. \end{cases} \tag{2}$$

$$P(A_{ij} = a|V_i = 0, V_j = *) = \begin{cases} P_0(A_{ij} = 1) & \text{if } a = 1, \\ P_0(A_{ij} = -1) & \text{if } a = -1, \\ P_0(A_{ij} = 0) & \text{if } a = 0. \end{cases} \tag{3}$$

where symbol $*$ is a "wildcard" character representing either 0 or 1, and $P_0(\cdot)$ represents the prior value on a given event.

This setup corresponds to an assumption that the states of the arc-adjacent nodes are insufficient to increase the certainty that the nodes interact. We are not altering the probability that two nodes have connection *no effect*, but can informatively re-distribute probability between *activation* and *inhibition*.

An assumption common across versions is that if the upstream node, V_i for an arc, A_{ij}, is inactive($V_i = 0$), the value for A_{ij} is sampled from the prior distribution for the arc, $P(A_{ij} = a_{ij})$. An inactive upstream node (V_i) does not provide evidence on its "effects" on the downstream node (V_j).

1.2 Arcs update version B

$$P(A_{ij} = a|V_i = 1, V_j = 1) = \begin{cases} 1 & \text{if } a = 1, \\ 0 & \text{if } a = -1, \\ 0 & \text{if } a = 0. \end{cases} \tag{4}$$

$$P(A_{ij} = a|V_i = 1, V_j = 0) = \begin{cases} 0 & \text{if } a = 1, \\ 1 & \text{if } a = -1, \\ 0 & \text{if } a = 0. \end{cases} \tag{5}$$

$$P(A_{ij} = a | V_i = 0, V_j = *) = \begin{cases} P_0(A_{ij} = 1) & \text{if } a = 1, \\ P_0(A_{ij} = -1) & \text{if } a = -1, \\ P_0(A_{ij} = 0) & \text{if } a = 0. \end{cases} \tag{6}$$

This version corresponds to an assumption that the states of the arc-adjacent nodes can be used as direct evidence in support of existence of an interaction.

1.3 Arcs update version C

Consider the conditional probability

$$P(A_{ij} = a_{ij} | V_i = v_i, V_j = v_j) = \frac{P(a_{ij}, v_i, v_j)}{P(v_i, v_j)} \propto p(a_{ij}|v_i)p(v_j|a_{ij}, v_i). \tag{7}$$

To simplify the computation, we assume that A_{ij} is independent from the incoming node V_i and we only consider inputs from V_i and A_{ij} when deciding the output at V_j. Then, from (7), the conditional probabilities for updating A_{ij} are derived as follows:

$$P(A_{ij} = a | V_i = 1, V_j = 1) = \begin{cases} \dfrac{P_0(A_{ij} = 1)}{P_0(A_{ij} = 1) + P_0(A_{ij} = 0)P_0(V_j = 1)} & \text{if } a = 1, \\ 0 & \text{if } a = -1, \\ \dfrac{P_0(A_{ij} = 0)P_0(V_j = 1)}{P_0(A_{ij} = 1) + P_0(A_{ij} = 0)P_0(V_j = 1)} & \text{if } a = 0. \end{cases} \tag{8}$$

$$P(A_{ij} = a | V_i = 1, V_j = 0) = \begin{cases} 0 & \text{if } a = 1, \\ \dfrac{P_0(A_{ij} = -1)}{P_0(A_{ij} = -1) + P_0(A_{ij} = 0)P_0(V_j = 0)} & \text{if } a = -1, \\ \dfrac{P_0(A_{ij} = 0)P_0(V_j = 0)}{P_0(A_{ij} = -1) + P_0(A_{ij} = 0)P_0(V_j = 0)} & \text{if } a = 0. \end{cases} \tag{9}$$

$$P(A_{ij} = a | V_i = 0, V_j = *) = \begin{cases} P_0(A_{ij} = 1) & \text{if } a = 1, \\ P_0(A_{ij} = -1) & \text{if } a = -1, \\ P_0(A_{ij} = 0) & \text{if } a = 0. \end{cases} \tag{10}$$

This update uses Bayesian probability calculation and allows for a more intuitive redistribution of probabilities. It can also be readily extended to more sophisticated updating schemes by incorporating more complex models for multiple inputs at V_j.

1.4 Arcs update version D

A slightly modified version.

$$P(A_{ij} = a | V_i = 1) \propto \begin{cases} P_0(A_{ij} = 1)/P_0(V_i = 1) & a = 1 \\ P_0(A_{ij} = -1)/P_0(V_i = 1) & a = -1 \\ P_0(A_{ij} = 0) & a = 0 \end{cases}$$

$$P(A_{ij}=a|V_i=1,V_j=1)=\begin{cases}\dfrac{P_0(A_{ij}=1)}{P_0(A_{ij}=1)+P_0(A_{ij}=0)P_0(V_i=1)P_0(V_j=1)} & \text{if } a=1,\\ 0 & \text{if } a=-1,\\ \dfrac{P_0(A_{ij}=0)P_0(V_i=1)P_0(V_j=1)}{P_0(A_{ij}=1)+P_0(A_{ij}=0)P_0(V_i=1)P_0(V_j=1)} & \text{if } a=0.\end{cases} \tag{11}$$

$$P(A_{ij}=a|V_i=1,V_j=0)=\begin{cases}0 & \text{if } a=1,\\ \dfrac{P_0(A_{ij}=-1)}{P_0(A_{ij}=-1)+P_0(A_{ij}=0)P_0(V_i=1)P_0(V_j=0)} & \text{if } a=-1,\\ \dfrac{P_0(A_{ij}=0)P_0(V_i=1)P_0(V_j=0)}{P_0(A_{ij}=-1)+P_0(A_{ij}=0)P_0(V_i=1)P_0(V_j=0)} & \text{if } a=0.\end{cases} \tag{12}$$

$$P(A_{ij}=a|V_i=0,V_j=*)=\begin{cases}P_0(A_{ij}=1) & \text{if } a=1,\\ P_0(A_{ij}=-1) & \text{if } a=-1,\\ P_0(A_{ij}=0) & \text{if } a=0.\end{cases} \tag{13}$$

This arc update version differs in that when there is an input from V_i (i.e., $V_i=1$), the arc action likelihood is updated. The alteration maintains the ratio of the probabilities on *activation* and *inhibition*, and down-weights the probability on *no effect* by an artificial factor $P_0(V_i=1)$.

1.5 Arcs update: Version E

One last proposal; a more generalized of version D.

$$P(A_{ij}=a|V_i=1,V_j=1)=\begin{cases}\dfrac{P_0(A_{ij}=1)}{P_0(A_{ij}=1)+\beta P_0(A_{ij}=0)P_0(V_i=1)P_0(V_j=1)} & \text{if } a=1,\\ 0 & \text{if } a=-1,\\ \dfrac{P_0(A_{ij}=0)P_0(V_i=1)P_0(V_j=1)}{P_0(A_{ij}=1)+\beta P_0(A_{ij}=0)P_0(V_i=1)P_0(V_j=1)} & \text{if } a=0.\end{cases} \tag{14}$$

$$P(A_{ij}=a|V_i=1,V_j=0)=\begin{cases}0 & \text{if } a=1,\\ \dfrac{P_0(A_{ij}=-1)}{P_0(A_{ij}=-1)+\beta P_0(A_{ij}=0)P_0(V_i=1)P_0(V_j=0)} & \text{if } a=-1,\\ \dfrac{P_0(A_{ij}=0)P_0(V_i=1)P_0(V_j=0)}{P_0(A_{ij}=-1)+\beta P_0(A_{ij}=0)P_0(V_i=1)P_0(V_j=0)} & \text{if } a=0.\end{cases} \tag{15}$$

$$P(A_{ij} = a | V_i = 0, V_j = *) = \begin{cases} P_0(A_{ij} = 1) & \text{if } a = 1, \\ P_0(A_{ij} = -1) & \text{if } a = -1, \\ P_0(A_{ij} = 0) & \text{if } a = 0. \end{cases} \quad (16)$$

Here the probability on *no effect* is down-weighted by a factor $\beta P_0(V_i = 1)$ where an arbitrary ($0 \leq \beta \leq 1$) can be named. When $\beta = 0$) this equation is equivalent to version C. When $\beta = 1$) this equation is equivalent to version D.

1.6 Multiple arcs connected to the same node

.

The scenario gets more complicated when you consider a situation where there are multiple adjacent arcs to one node. One possible way to proceed is to update each arc independently (as outlined above).We favor the simplicity of this solution. The one by one idea behind our "independent arc update" is in accordance with the way in which most interactions are discovered and published in the literature [1]. Since these interactions are independently derived, they can be tested for consistency with node-related data in the same fashion.

An alternate solution would be to update values for all the arcs adjacent to the same node *simultaneously.* We are not addressing this in detail in this article because of the difficulty of finding a set of assumptions that we can plausibly justify by biological data. Another complication of multiple-arc updating is the possibility of parental nodes to the given node which are not included in the data. Such a complicated update would not give good probability estimates while adding computational burden.

[1]A notable exception is associated with so-called high-throughput experiments, such as yeast two-hybrid experiments aiming at discovering protein-protein interactions.

2 Supplement B: updating nodes given fixed arcs

In this section we describe different options for updating a node given fixed incoming arcs. Our topology is a directed graph to incorporate causal knowledge [2]. This allows us to calculate the joint distribution for all nodes by decomposing the computation into the product of the conditional probabilities of nodes, their parental nodes and associated arcs.

We begin with the input nodes (nodes with no parent nodes) and follow the graph until all sink nodes (nodes with no offspring nodes) are updated.

$$G \longrightarrow B \longrightarrow C \longrightarrow D.$$

Node G is the only input node, and node D is the only sink node. The joint distribution of node values given arcs for this simple graph has the following form:

$$\begin{aligned} P(V_G &= v_G, V_B = v_B, V_C = v_C, V_D = v_D | A_{GB} = a_{GB}, A_{BC} = a_{BC}, A_{CD} = a_{CD}) \\ &= P(V_G = v_G) P(V_B = v_B | V_G = v_G, A_{GB} = a_{GB}) \\ &\times P(V_C = v_C | V_B = v_B, A_{BC} = a_{BC}) \\ &\times P(V_D = v_D | V_C = v_C, A_{CD} = a_{CD}). \end{aligned} \tag{17}$$

Therefore, to arrive at a sample from the full joint distribution of nodes given arcs, we first update the value for the input node (G)—sampling from its prior distribution. For all non-input nodes (B, C, and D) the value is sampled from the appropriate conditional distributions (given the values of arcs and parental nodes for the node being updated). Each child node can be updated only after all parental nodes are updated.

A more general case of node updating concerns sampling of values for a node, V, that has multiple incoming edges with possibly conflicting arc values (both *inhibition* and *activation* values). We need to define the conditional probability that $V_i = v_i$ given the values of upstream nodes and arcs—assuming that node V_i has n parents, with values $\{V_j = v_j\}_{j=1,\ldots,n}$ at the parental nodes, and the values $\{A_{ij} = a_{ij}\}_{j=1,\ldots,n}$ at the direct incoming arcs of V_i. Different definitions of this conditional probability actually represent different assumptions and models at the local network of V_j.

2.1 Node update version A

$$P\left(V_i = 1 \middle| \begin{matrix} \{V_j = v_j\}_{j=1,\ldots,n} \\ \{A_{ij} = a_{ij}\}_{j=1,\ldots,n} \end{matrix}, j \in par(V_i)\right) = \begin{cases} \phi(I_i^+ \rhd I_i^-) & \text{if } I_i^+ > 0, \text{and } I_i^- > 0 \\ 1 & \text{if } I_i^+ > 0, \text{and } I_i^- = 0 \\ 0 & \text{if } I_i^+ = 0, \text{and } I_i^- > 0 \\ P_0(V_i = 1) & \text{if } I_i^+ = 0, \text{and } I_i^- = 0 \end{cases} \tag{18}$$

[2] This first version is a directed acyclic graph - we hope to handle cycles in the next version.

$$P\left(V_i=0 \middle| \begin{array}{l}\{V_j=v_j\}_{j=1,\dots,n}\\ \{A_{ij}=a_{ij}\}_{j=1,\dots,n}\end{array}, j\in par(V_i)\right) = 1-P\left(V_i=1 \middle| \begin{array}{l}\{V_j=v_j\}_{j=1,\dots,n}\\ \{A_{ij}=a_{ij}\}_{j=1,\dots,n}\end{array}, j\in par(V_i)\right) \tag{19}$$

where $I_i^+ = \sum_{\{j:\, a_{ij}=1\}} v_j$ and $I_V^- = \sum_{\{j:\, a_{ij}=-1\}} v_j$ are combined incoming activating and inhibiting signals upon V_i. Here $\phi(I_i^+ \triangleright I_i^-)$ is a function that resolves a conflict between activating and inhibiting signals—in the simplest case it can be a single probability parameter common for the whole network. In a more sophisticated setup, the value of the parameter can vary for different nodes. In even more complex case, function $\phi(I_i^+ \triangleright I_i^-)$ can explicitly depend on values of I_i^+ and I_i^- and have different a form for different nodes in the network.

2.2 Node update version B

$$P\left(V_i=1 \middle| \begin{array}{l}\{V_j=v_j\}_{j=1,\dots,n}\\ \{A_{ij}=a_{ij}\}_{j=1,\dots,n}\end{array}, j\in par(V_i)\right) = \begin{cases} P_0(V_i=1) & \text{if } I_i^+>0, \text{and } I_i^->0\\ 1 & \text{if } I_i^+>0, \text{and } I_i^-=0\\ 0 & \text{if } I_i^+=0, \text{and } I_i^->0\\ P_0(V_i=1) & \text{if } I_i^+=0, \text{and } I_i^-=0\end{cases} \tag{20}$$

$$P\left(V_i=0 \middle| \begin{array}{l}\{V_j=v_j\}_{j=1,\dots,n}\\ \{A_{ij}=a_{ij}\}_{j=1,\dots,n}\end{array}, j\in par(V_i)\right) = 1-P\left(V_i=1 \middle| \begin{array}{l}\{V_j=v_j\}_{j=1,\dots,n}\\ \{A_{ij}=a_{ij}\}_{j=1,\dots,n}\end{array}, j\in par(V_i)\right) \tag{21}$$

This update version resolves conflicting inputs by an assumption that every conflict in input signals brings us back to the prior distribution for the node.

2.3 Node update version C

In this version, we assume that state of node V_i is determined by a Boolean function over values of $V_j \in par(V_i)$ and values of the corresponding edges, A_{ij}. For example, if vertex V_1 has just two parental nodes, V_2 and V_3, as shown below:

$$\begin{array}{ccc} V_2 & & V_3\\ & \searrow \quad \swarrow & \\ & V_1 & \end{array} \tag{22}$$

we can assume that node V_1 is active *only* if both parental nodes are active, $V_2 = V_3 = 1$, and both arcs are in the state *activation*, $A_{21} = A_{31} = 1$. Similarly, we can assume that to inhibit V_1 we need both incoming edges in the state *inactivation*, $A_{21} = A_{31} = -1$, and both parental nodes active. Then the probability to find node V_1 activated given states of parental nodes and arc is as follows.

$$P\left(V_1 = 1 \middle| \begin{array}{c} V_2 = v_2, V_3 = v_3 \\ A_{21} = a_{21}, A_{31} = a_{31} \end{array}\right) = \begin{cases} \mathbf{1} & \text{if } (v_2, v_3, a_{21}, a_{31}) = (1,1,1,1), \\ 0 & \text{if } (v_2, v_3, a_{21}, a_{31}) = (1,1,-1,-1), \\ P_0(V_1 = 1) & \text{otherwise.} \end{cases} \tag{23}$$

Given n inputs to a node V_i, we can define the state of the node as a probabilistic function of all possible $2^n \times 3^n$ states of the parental nodes and arcs. Unfortunately, it would be very hard to find biological data to estimate parameters for such modeling at the present state of knowledge about biological circuitry.

3 Supplement C: enumeration examples

In this section, we give numerical illustrations of different arc update and node update versions. For comparison, in addition to the arc updates version considered in supplement A, we also include examples where the arcs are assumed to be independent with the nodes.

3.1 Independent arcs

Consider a special case when distribution values of the arcs are fixed despite the values of incoming nodes, that is,

$$P(\mathbf{A}|\mathbf{V}) = P(\mathbf{A}) = \prod_{j,i} P(A_{ij} = a_{ij}). \tag{24}$$

Then, we can compute the marginal probabilities for nodes through direct summation over the appropriate joint distribution for nodes and arcs. Below we illustrate computation of the joint and marginal distributions for a simple linear network example.

3.2 Linear example

$$G \longrightarrow B \longrightarrow C \longrightarrow D. \tag{25}$$

Priors for the nodes:
$P_0(V_G = 1) = 0.9$, $P_0(V_G = 0) = 0.1$,
$P_0(V_B = 1) = 0.9$, $P_0(V_B = 0) = 0.1$,
$P_0(V_C = 1) = 0.9$, $P_0(V_C = 0) = 0.1$,
$P_0(V_D = 1) = 0.9$, $P_0(V_C = 0) = 0.1$.

Priors for arcs:
$P_0(A_{GB} = [1, -1, 0]) \rightarrow (0.9, 0.05, 0.05)$
$P_0(A_{BC} = [1, -1, 0]) \rightarrow (0.05, 0.9, 0.05)$
$P_0(A_{CD} = [1, -1, 0]) \rightarrow (0.7, 0.2, 0.1)$.

For this simple example, the exact enumeration of the joint probabilities and marginal probabilities with independent arcs may be carried out as follows.

$$\begin{aligned} P(V_G &= v_G, V_B = v_B, V_C = v_C, V_D = v_D, A_{GB} = a_{GB}, A_{BC} = a_{BC}, A_{CD} = a_{CD}) \\ &= P_0(V_G = v_G) P_0(A_{GB} = a_{GB}) P(V_B = v_B | V_G = v_G, A_{GB} = a_{GB}) \\ &\times P_0(A_{BC} = a_{BC}) P(V_C = v_C | V_B = v_B, A_{BC} = a_{BC}) \\ &\times P_0(A_{CD} = a_{CD}) P(V_D = v_D | V_C = v_C, A_{CD} = a_{CD}). \end{aligned} \tag{26}$$

$$\begin{aligned} P(V_G = v_G) \stackrel{\text{def}}{=} & \sum_{v_B=0,1} \sum_{v_C=0,1} \sum_{v_D=0,1} \sum_{a_{GB}=-1,0,1} \sum_{a_{BC}=-1,0,1} \\ & \sum_{a_{CD}=-1,0,1} P(V_G = v_G, V_B = v_B, \\ & V_C = v_c, V_D = v_D, A_{GB} = a_{GB}, \\ & A_{BC} = a_{BC}, A_{CD} = a_{CD}). \end{aligned} \tag{27}$$

$$P(A_{ij} = a_{ij}) \equiv P_0(A_{ij} = a_{ij}) \text{ (prior values) }, \ \forall i, j. \tag{28}$$

Enumerated marginals for the nodes can be easily calculated:
$P(V_G = 1) = 0.9$, $P(V_G = 0) = 0.1$,
$P(V_B = 1) = 0.9405$, $P(V_B = 0) = 0.0595$,
$P(V_C = 1) = 0.1429$, $P(V_C = 0) = 0.8571$,
$P(V_D = 1) = 0.8843$, $P(V_D = 0) = 0.1157$.

3.3 X-shaped example

In this section, we use a more general example to illustrate different arc and node update versions, starting with simpler computations where independent arcs are assumed.

$$\begin{array}{ccccc} G & & & & B \\ & \searrow & & \swarrow & \\ & & C & & \\ & \swarrow & & \searrow & \\ D & & & & E \end{array} \tag{29}$$

Priors for the nodes:
$P_0(V_G = 1) = 0.7$, $P_0(V_G = 0) = 0.3$,
$P_0(V_B = 1) = 0.8$, $P_0(V_B = 0) = 0.2$,
$P_0(V_C = 1) = 0.9$, $P_0(V_C = 0) = 0.1$,
$P_0(V_D = 1) = 0.35$, $P_0(V_D = 0) = 0.65$,

$P_0(V_E = 1) = 0.75,\ P_0(V_E = 0) = 0.25.$

Priors for arcs:
$P_0(A_{GC} = [1, -1, 0]) \to (0.9, 0.05, 0.05),$
$P_0(A_{BC} = [1, -1, 0]) \to (0.05, 0.85, 0.1),$
$P_0(A_{CD} = [1, -1, 0]) \to (0.8, 0.11, 0.09),$
$P_0(A_{CE} = [1, -1, 0]) \to (0.1, 0.1, 0.8).$

3.3.1 Independent arcs

Assuming independent arcs, the probabilities can be enumerated as follows.

$$\begin{aligned}
P(V_G &= v_G, V_B = v_B, V_C = v_C, V_D = v_D, V_E = v_E, \\
A_{GC} &= a_{GC}, A_{BC} = a_{BC}, A_{CD} = a_{CD}, A_{CE} = a_{CE}) \\
&= P_0(V_G = v_G) P_0(A_{GC} = a_{GC}) \\
&\times P_0(V_B = v_B) P_0(A_{BC} = a_{BC}) \\
&\times P(V_C = v_C | V_G = v_G, V_B = v_B, A_{GC} = a_{GC}, A_{BC} = a_{BC}) \\
&\times P_0(A_{CD} = a_{CD}) P(V_D = v_D | V_C = v_C, A_{CD} = a_{CD}) \\
&\times P_0(A_{CE} = a_{CE}) P(V_E = v_E | V_C = v_C, A_{CE} = a_{CE}).
\end{aligned} \tag{30}$$

We implemented this example with two of our node update version. Table 1 shows the results for using **node update version A** with $\phi(I_i^+ \rhd I_i^-) = \frac{1}{2}$ (Equation (18)). The enumerated marginals (using exact probability computation) on the nodes are compared with both the priors and the caluculated values using the proposed numerical updates with Gibbs sampler. Since we are assuming independent arcs for this computation, the marginals on the arcs are unchanged from the prior values. The resulting network is compared with the network based on prior values in Figure 1.

Node	*Priors*		*Exact marginals*		*Gibbs sampler*	
	$v = 1$	$v = 0$	$v = 1$	$v = 0$	$v = 1$	$v = 0$
V_G	0.7	0.3	0.7	0.3	0.6996	0.30048
V_B	0.8	0.2	0.8	0.2	0.7999	0.2001
V_C	0.9	0.1	0.5143	0.4857	0.5140	0.4860
V_D	0.35	0.65	0.5976	0.4024	0.5974	0.4026
V_E	0.75	0.25	0.7243	0.2757	0.7243	0.2757

Table 1: Marginals for the nodes exact and estimated with the Gibbs sampler: node update version A. (Gibbs sampler: $50,000$ different starting points, 100 iterations for each chain.)

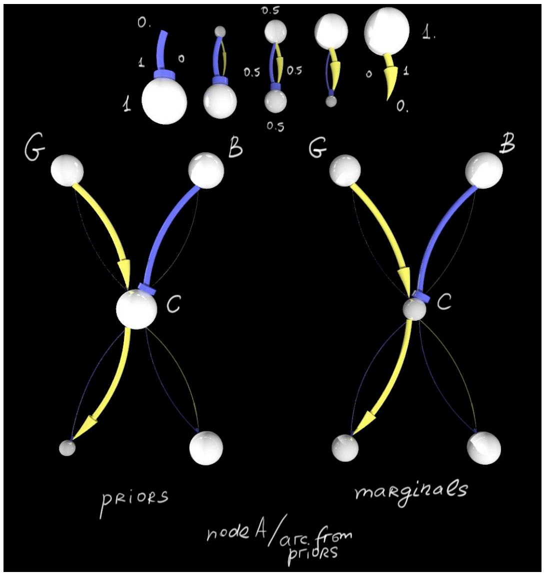

Figure 1: Update of both arcs and nodes of a hypothetical five-node and four-arc network (node update version A, arc update from priors).

For comparison, we also implemented the enumeration and numerical updating using the **node update version B**. The results are listed in Table 2.

Node	*Priors*		*Exact marginals*		*Gibbs sampler*	
	$v = 1$	$v = 0$	$v = 1$	$v = 0$	$v = 1$	$v = 0$
V_G	0.7	0.3	0.7	0.3	0.7002	0.2998
V_B	0.8	0.2	0.8	0.2	0.8001	0.1999
V_C	0.9	0.1	0.6862	0.3138	0.6864	0.3136
V_D	0.35	0.65	0.6804	0.3196	0.6802	0.3198
V_E	0.75	0.25	0.7157	0.2843	0.7156	0.2844

Table 2: Marginals for the nodes—comparison of the exact computation with the Gibbs sampler: node update version B.(Gibbs sampler: $50,000$ different starting points, 100 iterations for each chain.)

Apparently from Tables 1 and 2, the Gibbs sampler gives acceptable estimates of the marginals (precision can be improved by increasing the number of sampling iterations).

These examples also illustrate the difference between node update versions A and B. In the case of conflicting inputs for node C, version A assumes complete lack of information about the state of V_c ($\phi(I_i^+ \rhd I_i^-) = \frac{1}{2}$, Equation 18), while version B samples values from the prior distribution for node V_C.

3.3.2 Updating both nodes and arcs

In this section, we apply different versions of arc and node update combinations to the same numerical x-shape example, with exactly the same prior values.

Node update	Arc update	Numerical results	Graph
Version A	Version C	Tables 4 and 5	Figure 2
Version A	Version D	Tables 6 and 7	Figure 3
Version B	Version B	Tables 8 and 9	Figure 4
Version B	Version C	Tables 10 and 11	Figure 5
Version B	Version E (with $\beta = 0.2$)	Tables 12 and 13	Figure 6

Table 3: Versions of update depicted in the supplement.

Node	*Priors*		*Marginals (Gibbs sampler)*	
	$v = 1$	$v = 0$	$v = 1$	$v = 0$
V_G	0.7000	0.3000	0.7005	0.2995
V_B	0.8000	0.2000	0.8000	0.2000
V_C	0.9000	0.1000	0.5147	0.4853
V_D	0.3500	0.6500	0.5979	0.4021
V_E	0.7500	0.2500	0.7243	0.2757

Table 4: Marginals for the nodes estimated with the Gibbs sampler: node update model A, arc update model C. (Gibbs sampler: $10,000$ different starting points, 100 iterations for each chain.)

Arc	*Priors*			*Marginals (Gibbs sampler)*		
	$a=1$	$a=-1$	$a=0$	$a=1$	$a=-1$	$a=0$
A_{GC}	0.9000	0.0500	0.0500	0.8998	0.0500	0.0501
A_{BC}	0.0500	0.8500	0.1000	0.0499	0.8499	0.1002
A_{CD}	0.8000	0.1100	0.0900	0.8007	0.1096	0.0897
A_{CE}	0.1000	0.1000	0.8000	0.1002	0.0999	0.7999

Table 5: Marginals for the arcs estimated with the Gibbs sampler: node update model A, arc update model C. (Gibbs sampler: 10,000 different starting points, 100 iterations for each chain.)

Node	*Priors*		*Marginals (Gibbs sampler)*	
	$v=1$	$v=0$	$v=1$	$v=0$
V_G	0.7000	0.3000	0.6994	0.3006
V_B	0.8000	0.2000	0.8004	0.1996
V_C	0.9000	0.1000	0.5762	0.4238
V_D	0.3500	0.6500	0.6215	0.3785
V_E	0.7500	0.2500	0.7199	0.2801

Table 6: Marginals for the nodes estimated with the Gibbs sampler: node update model A, arc update model D. (Gibbs sampler: 10,000 different starting points, 100 iterations for each chain.)

4 Supplement D. Graph support

In this section, we included the data supporting the graph depicting the genes assoiciated with the four neurological diseases.

4.1 List of Nodes

4.2 List of Arcs

4.3 List of Arc Types

4.4 List of Brain Tissue Names

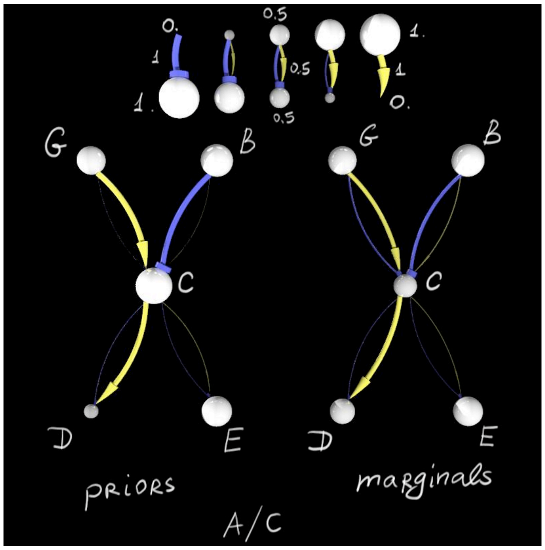

Figure 2: Update of both arcs and nodes of a hypothetical five-node and four-arc network (node update version A, arc update version C—detailed numerical information corresponding to this figure is shown in Tables 4 and 5).

Arc	*Priors*			*Marginals (Gibbs sampler)*		
	$a = 1$	$a = -1$	$a = 0$	$a = 1$	$a = -1$	$a = 0$
A_{GC}	0.9000	0.0500	0.0500	0.6766	0.2757	0.0477
A_{BC}	0.0500	0.8500	0.1000	0.1833	0.5438	0.2729
A_{CD}	0.8000	0.1100	0.0900	0.7941	0.1179	0.0880
A_{CE}	0.1000	0.1000	0.8000	0.1056	0.1046	0.7898

Table 7: Marginals for the arcs estimated with the Gibbs sampler: node update model A, arc update model D. (Gibbs sampler: $10,000$ different starting points, 100 iterations for each chain.)

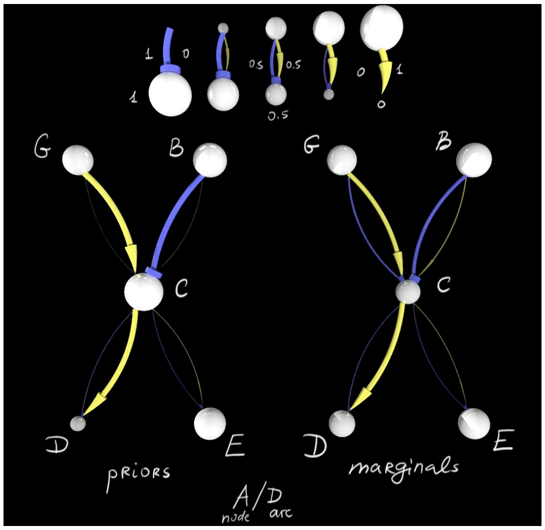

Figure 3: Update of both arcs and nodes of a hypothetical five-node and four-arc network (node update version A, arc update version D (equivalent to version E with $\beta = 1$)—detailed numerical information corresponding to this figure is shown in Tables 6 and 7).

Node	*Priors*		*Marginals (Gibbs sampler)*	
	$v = 1$	$v = 0$	$v = 1$	$v = 0$
V_G	0.7	0.3	0.6999	0.3001
V_B	0.8	0.2	0.8000	0.2000
V_C	0.9	0.1	0.8455	0.1545
V_D	0.35	0.65	0.7241	0.3199
V_E	0.75	0.25	0.7105	0.2895

Table 8: Marginals for the nodes estimated with the Gibbs sampler: node update model B, arc update model B.(Gibbs sampler: $50,000$ different starting points, 100 iterations for each chain.)

Arc	*Priors*			*Marginals (Gibbs sampler)*		
	$a=1$	$a=-1$	$a=0$	$a=1$	$a=-1$	$a=0$
A_{GC}	0.9	0.05	0.05	0.8962	0.0887	0.0151
A_{BC}	0.05	0.85	0.1	0.6745	0.3055	0.02
A_{CD}	0.8	0.11	0.09	0.7913	0.1948	0.0139
A_{CE}	0.1	0.1	0.8	0.61052	0.2662	0.1233

Table 9: Marginals for the arcs estimated with the Gibbs sampler: node update model B, arc update model B. (Gibbs sampler: 50,000 different starting points, 100 iterations for each chain.)

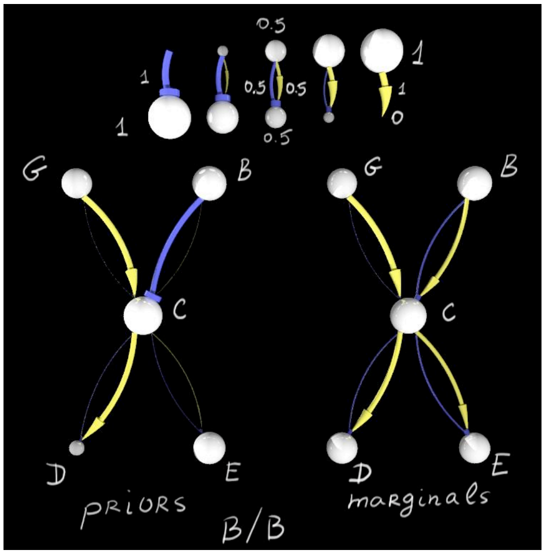

Figure 4: Update of both arcs and nodes of a hypothetical five-node and four-arc network (node update version B, arc update version B—detailed numerical information corresponding to this figure is shown in Tables 8 and 9).

Node	*Priors*		*Marginals (Gibbs sampler)*	
	$v = 1$	$v = 0$	$v = 1$	$v = 0$
V_G	0.7000	0.3000	0.7001	0.2999
V_B	0.8000	0.2000	0.7998	0.2002
V_C	0.9000	0.1000	0.8057	0.1943
V_D	0.3500	0.6500	0.7306	0.2694
V_E	0.7500	0.2500	0.7102	0.2898

Table 10: Marginals for the nodes estimated with the Gibbs sampler: node update model B, arc update model C. (Gibbs sampler: $10,000$ different starting points, 100 iterations for each chain.)

Arc	*Priors*			*Marginals (Gibbs sampler)*		
	$a = 1$	$a = -1$	$a = 0$	$a = 1$	$a = -1$	$a = 0$
A_{GC}	0.9000	0.0500	0.0500	0.8455	0.1023	0.0522
A_{BC}	0.0500	0.8500	0.1000	0.2339	0.3411	0.4249
A_{CD}	0.8000	0.1100	0.0900	0.7911	0.1161	0.0928
A_{CE}	0.1000	0.1000	0.8000	0.1001	0.0999	0.7999

Table 11: Marginals for the arcs estimated with the Gibbs sampler: node update model B, arc update model C. (Gibbs sampler: $10,000$ different starting points, 100 iterations for each chain.)

Node	*Priors*		*Marginals (Gibbs sampler)*	
	$v = 1$	$v = 0$	$v = 1$	$v = 0$
V_G	0.7000	0.3000	0.6999	0.3001
V_B	0.8000	0.2000	0.8001	0.1999
V_C	0.9000	0.1000	0.8331	0.1669
V_D	0.3500	0.6500	0.7441	0.2559
V_E	0.7500	0.2500	0.6454	0.3546

Table 12: Marginals for the nodes estimated with the Gibbs sampler: node update model B, arc update model E (with $\beta = 0.2$). (Gibbs sampler: $10,000$ different starting points, 100 iterations for each chain.)

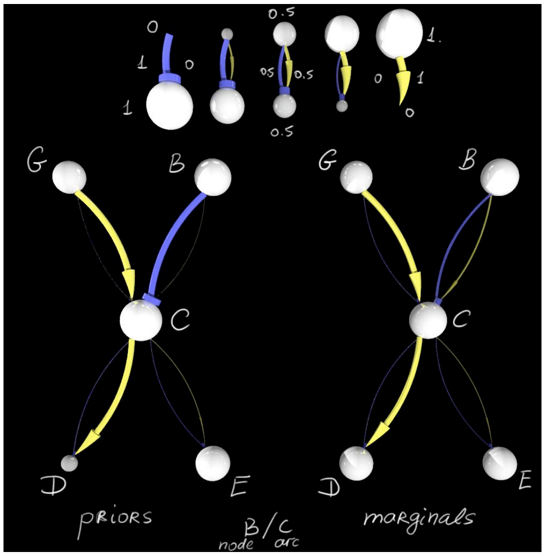

Figure 5: Update of both arcs and nodes of a hypothetical five-node and four-arc network (node update version B, arc update version C—detailed numerical information corresponding to this figure is shown in Tables 10 and 11).

Arc	*Priors*			*Marginals (Gibbs sampler)*		
	$a = 1$	$a = -1$	$a = 0$	$a = 1$	$a = -1$	$a = 0$
A_{GC}	0.9000	0.0500	0.0500	0.8854	0.0941	0.0204
A_{BC}	0.0500	0.8500	0.1000	0.5173	0.3168	0.1659
A_{CD}	0.8000	0.1100	0.0900	0.8123	0.1549	0.0329
A_{CE}	0.1000	0.1000	0.8000	0.2672	0.2462	0.4867

Table 13: Marginals for the arcs estimated with the Gibbs sampler: node update model B, arc update model E (with $\beta = 0.2$), (Gibbs sampler: $10,000$ different starting points, 100 iterations for each chain.)

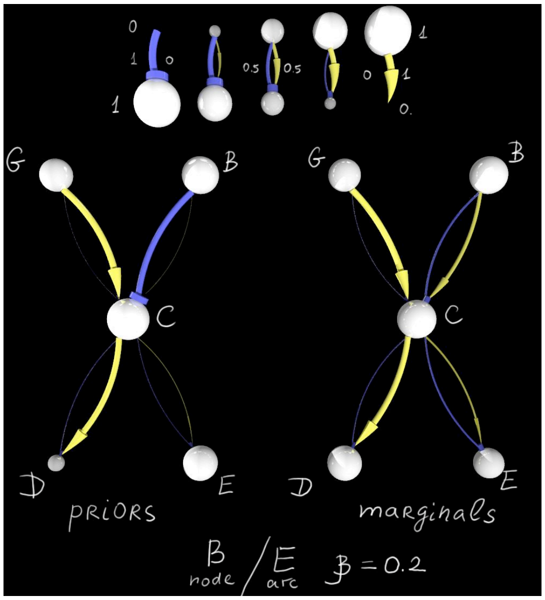

Figure 6: Update of both arcs and nodes of a hypothetical five-node and four-arc network (node update version B, arc update version E (with $\beta = 0.2$)—detailed numerical information corresponding to this figure is shown in Tables 12 and 13).
