# Supplementary material for: Self-Correcting Maps of Molecular Pathways
Source: PLoS One. 2006 Dec 20;1(1):e61. doi: 10.1371/journal.pone.0000061 (PMC1762305; doi:10.1371/journal.pone.0000061)
Supplement: Supplement S2 — Additional information about dataset used (0.61 MB PDF) [file pone.0000061.s002.pdf]

Supplemental tables of support for the graph.

D.1 List of Nodes

This table is a list of all of the nodes which are in the graph. For each node there are seven columns of information. The second column identifies those nodes which we associated with diseases on the graph. The third column highlights the diseases which OMIM identifies for each gene. The fourth column is the entrez gene id that is associated with each node value. When more than one entrez gene id is listed it represents a gene name that is used as a synonym for more than one gene. The last 2 columns have the HGNC ID and miscellaneous notes.

GENE SYMBOL	NODE DISEASE	DISEASE FROM OMIM	ENTREZ GENE ID	SYNONYM	HGNC	NOTES
abl1		Leukemia, Philadelphia chromosome-positive	25	abl<br>c-abl<br>cabl<br>v-abl	76	
aes			166		307	
ahr			196		348	
akap5			9495	akap79	375	
akt1	Schizo	Schizophrenia	207	akt-1	391	
ampk			5562-prkaa1<br>5563-prkaa2<br>5564-prkab1		9376<br>9377<br>9378	All subunits are collapsed up into the one alias ampk
ang			283		483	
ap-2			7020 –tfap2a	ap-2 is an alias for tfap2a	11742	
ap3			1176 – ap3s1<br>8120 – ap3b2<br>8546 – ap3b1<br>8943 – ap3d1<br>10239 – ap3s2<br>10947 – ap3m2<br>26985 – ap3m1		2013<br>567<br>566<br>568<br>571<br>570<br>569	All subunits are collapsed up into the one alias ap3
apaf1			317		576	
apbb1			322	fe65	581	
apc		Adenoma, periampullary Adenomatous polyposis coli Colorectal cancer, Desmoid disease, hereditary, Gardner syndrome Gastric cancer,	324	gs	583	

		Turcot syndrome,				
apoe	Alz	Alzheimer disease-2<br>Hyperlipoproteinemia, type III<br>Sea-blue histiocyte disease<br>Myocardial infarction susceptibility	348	apoplipoprotein e	613	
apoer2			7804 lrp8	apoer2 is an alias for lrp8	6700	
app	Alz	Alzheimer disease-1, APP-related Amyloidosis, cerebroarterial, Iowa type	351	ad1<br>abeta	620	
arnt		Leukemia, acute myeloblastic	405	aryl hydrocarbon receptor nuclear translocator	700	
atf3			467		785	
atf5			22809		790	
atf6			22926		791	
atp8a2			51761	atp	13533	
atr		Seckel syndrome 1	545 - atr	84168 – antxr1	882	
baat		Hypercholanemia, familial	570	bt	570	
bace1			23621	bace<br>asp2	933	
bad			572		936	
bak1			578	bak	949	
bard1		Breast cancer, susceptibility to	580		952	
bax		Colorectal cancer T-cell acute lymphoblastic leukemia	581		959	
bccip			56647		978	
bche		Apnea, postanesthetic	590	e1	983	
bcl2		Leukemia/lymphoma, B-cell	596	bcl-2	990	
bcl2l1			598	bcl-x<br>bcl-xl<br>bcl-xs	992	
bcr		Leukemia, acute lymphocytic	613		1014	
bdnf		Central hypoventilation syndrome, congenital, Memory impairment, Obsessive-compulsive disorder, protection against	627		1033	
blmh	Alz	Alzheimer disease	642	bmh	1059	
blr1			643	cxcr5	1060	
bmp2			650	bone morphogenic preotein 2	1069	

bmp4			652		1071	
bnip3			664		1084	
brca1		Breast-ovarian cancer Papillary serous carcinoma of the peritoneum	672		1100	
brf1			677		1107	
calm1			801 – calm1<br>805 – calm2		1442<br>1445	All subunits are collapsed up into the one alias calmodulin
camk2g			818	cam kinase ii<br>camkii	1463	
cap1			10487		20040	
cas			9564 – bcar1	bcar1 an alias for cas “Crk-associated substrate” mentioned in text	971	
cas1			64921 – casd1	o-acetyltransferase	16014	
casp3			836	caspace 3	1504	
casp8		Autoimmune lymphoproliferative syndrome, type IIB Hepatocellular carcinoma, somatic	841	flice<br>mach<br>mch5<br>procaspase-8	1509	
cav1			857	caveolin 1	1527	
cav3		Cardiomyopathy, familial hypertrophic Creatine phosphokinase, elevated serum Muscular dystrophy, limb-girdle, type IC Myopathy, distal, with decreased caveolin 3 Rippling muscle disease	859	cavieolin 3	1529	
caveolin			857 – cav1<br>858 – cav2<br>859 – cav3	not specific could be any of the three	1527<br>1528<br>1529	
cbl			867	c-cbl	1541	
cblc			23624		15961	
ccl4			6351		10630	
ccnd1		Centrocytic lymphoma	595	cyclin d1	1582	

		Leukemia/lymphoma, chronic B-cell<br>Multiple myeloma<br>Parathyroid adenomatosis<br>von Hippel-Lindau disease<br>Colorectal cancer				
ccne2			9134	cyclin e2	1590	
ccs			9973		1613	
cd160			11126	nk1	17013	
cd28			940		1653	
cdc42			998		1736	
cdk2			1017		1771	
cdk2ap1			8099	p12doc-1	14002	
cdk5			1020		1774	
cdkn2a		Li Fraumeni syndrome,<br>Melanoma and neural system tumor syndrome<br>Melanoma, cutaneous malignant<br>Orolaryngeal cancer, multiple<br>Pancreatic cancer/melanoma syndrome	1029	arf<br>ink4a<br>p14	1787	
ced-4			9994-casp8ap2	ced-4 is an alias for casp8ap2	1510	
centg2	Autism **		116987	agap1	16922	
cfdp1			10428	p97	1873	
cftr		Congenital bilateral absence of vas deferens,<br>Cystic fibrosis<br>Sweat chloride elevation without CF<br>Hypertrypsinemia, neonatal<br>Pancreatitis, idiopathic	1080	c	1884	
chd3			1107		1918	
chd4			1108		1919	
chek1			1111	chk1	1925	
chrd			8646	cordin	1949	
cks1b			1163	cks1<br>ckshs1	19083	
clathrin			1211 - clta<br>1212 - cltb		2090<br>2091	All subunits are collapsed

			1213 - cltc		2092	up into the one alias clathrin
clk1			1195	sty	2068	
cls			1210		2089	
coh1		Cohen syndrome	157680 - vps13b	coh1 is an alias for vps13b	2183	
creb1			1385	creb	2345	
crk			1398	crkii	2362	
crkl			1399		2363	
cul1			8454		2551	
cul2			8453	cullin 2	2552	
cycs			54205	cytochrome c	19986	
dag1			1605	dag	2666	
dapk3			1613	zipk	2676	
daxx			1616		2681	
dp			5729 – ptgdr		9591	
dcn			1634	decorin	2705	
dlg1			1739	sap97	2900	
e2			26765<br>1489080 – human papillomavirus E2<br>1737 – dlat<br>7319 – ube2a<br>7320 – ube2b<br>11065 – ube2c		2896<br>12472<br>12473<br>15937	From the literature there was 3 separate uses for the E2 symbol disgnation. Barring splitting them up I incorpaorated them all up into one.
e3			1738 – dld<br>7337 – ube3a<br>89910 – ube3b<br>9690 – ube3c		2898<br>12496<br>13478<br>16803	True to a lesser extent for E3.
e2f1			1869	e2f-1	3113	
e2f4			1874	e2f-4	3118	
e2f5			1875		3119	
e-cadherin			999	cdh1 – e-cadherin (epithelial)	1748	
edf1			8721	mbf1	3164	

egf			1950		3229	
egfr			1956	erbb<br>erbb1	3236	
egln1			54583	sm-20	1232	
egr1			1958	ngfi-a<br>zif268	3238	
eif4e			1977	eukaryotic translation initiation factor 4e<br>cbp	3287	
eif4g2			1982		3297	
emx2		Schizencephaly	2018		3341	
ep300		Colorectal cancer, Rubinstein-Taybi syndrome	2033	P300	3373	
eps15			2060		3419	
eralpha			2099 – esr1	eralpha is an alias for esr1	3467	
erbb3			2065	her3	3431	
ern1			2081	ire1	3449	
exo1			9156		3511	
f2		Dysprothrombinemia<br>Hyperprothrombinemia	2147	pt	3535	
fadd			8772	mort1<br>fas associating protein	8772	
fas		Squamous cell carcinoma, burn scar-related, somatic<br>Autoimmune lymphoproliferative syndrome	355 - fas<br>2194 – fasn	apo1<br>cd95	11920	
fes			2242		3657	
flj10261			55107 – tmem16a	flj10261 is an alias for tmem16a	21625	
fn1		Ehlers-Danlos syndrome, type X	2335	fn	3778	
foxj1			2302	hfh4	3816	
frs2			10818	frs2alpha<br>snt	16971	
fut1		Bombay phenotype	2523	hsc<br>hh	4012	
fyn			2534	src related kinase	4037	
g72	Schizo	Schizophrenia	267012 – daoa	g72 is an alias for daoa	21191	
gab1			2549		4066	
gab2			9846		14458	
gc		Graves disease	2638	dbp	4187	
glur2			2891 – gria2	glur2 is an alias for both gria2	4572	

			2912 – grm2	and grm2	4594	
grap2			9402	mona	4563	
grb2			2885		4566	
grin2b			2904	nr2b	4586	
grip1			23426 – grip1		18708	
grm3	Schizo		2913	glur3	4595	
grp			2922		4605	
gsk-3			2931- gsk3a<br>2932– gsk3b		4616<br>4617	
gtf2i			2969	tf11-i	4659	
gtf2ird1			9569	gtf3	4661	
hgs			9146	hrs	4897	
hbp1			26959		23200	
hck			3055		4840	
hd		Huntington disease	3064	huntington	4851	
hdac1			3065	rpd3	4852	
hdac2			3066		4853	
hdac9			9734	hdac	14065	
hipk2			28996		14402	
hlf			3131	hepatic leukemia factor	4977	
hps1		Hermansky-Pudlak syndrome1	3257		5163	
hrmt1l2			3276- prmt1	prmt1 is the current name for hrmt1l2	5187	
hspa5			3309	bip	5238	
hspa8			3312	hsc70	5241	
hspd1		Spastic paraplegia-13	3329	hsp60	5261	
hspg2		Dyssegmental dysplasia, Silverman-Handmaker type Schwartz-Jampel syndrome, type 1	3339	plc	5273	
icos		ICOS deficiency	29851		5351	
igbp1		Corpus callosum, agenesis of, with mental retardation, ocular coloboma and micrognathia	3476	alpha4	5461	
igf1		Growth retardation with deafness and mental retardation due to IGF1 deficiency	3479	igf-i	5464	
igfbp1			3484		5469	
il10		Graft-versus-host disease, protection against	3586		5962	

		HIV-1, susceptibility to<br>Rheumatoid arthritis, progression of				
il18			3606		5986	
il5			3567		6016	
il8			3576		6025	
irf			3659 – irf1<br>3660 – irf2<br>3661 – irf3<br>3662 – irf4<br>3663 – irf5<br>3664 – irf6<br>3665 – irf7<br>3394 – irf8		6116<br>6117<br>6118<br>6119<br>6120<br>6121<br>6122<br>5358	
jun			3725	ap1	6204	
kcna4			3739	kv1.4	6222	
kl		Coronary artery disease	9365		6344	
krt14		Epidermolysis bullosa simplex	3861	k14	6416	
lef1			51176		6551	
lrp			4035 – lrp1		6692	
lta		Myocardial infarction, susceptibility to	4049	lt	6709	
lu		Blood group, Lutheran system	4059-bcam	lu is an alias for bcam	6722	
maea			10296	emp	13731	
mapk1			5594	erk<br>erk2<br>p38	6871	
mapk8			5599	jnk<br>jnk1	6881	
mapk8ip1		Diabetes mellitus, noninsulin-dependent	9479	ib1<br>jip1	6882	
mapt		Dementia, Pick disease-like<br>Dementia, frontotemporal, with parkinsonism<br>Pallidopontonigral degeneration<br>Supranuclear palsy, progressive atypical<br>Tauopathy and respiratory failure<br>Parkinson disease, late-onset	4137	tau	6893	

max			4149		6913	
mdc1			9656		21163	
mdm2		Accelerated tumor formation	4193	hdm2	6973	
mef2d			4209		6997	
mitf		Tietz syndrome Waardenburg syndrome/ocular albinism, digenic	4286		14334	
miz1			7709 – zbtb17	miz1 is an alias for zbtb17	12936	
mkl1		Megakaryoblastic leukemia, acute	57591	mal	14334	
mlana			2315	melana	7124	
mllt4			4301	af-6	7137	
msx2		Craniosynostosis, type 2 Parietal foramina 1	4488		7392	
myc		Burkitt lymphoma	4609		7553	
nab1			4664		7626	
nab2			4665		7627	
napa			8775	alpha snap	7641	
ncoa1			8648	src-1	7668	
ncoa2			10499	grip1<br>tif2	7669	
nedd8			4738		7732	
nes			10763		7756	
nf-kappab			4790 - nfkb1	as a descriptor for nfkb1 nf kappab is an alias	7794	
nfatc1			4772	nfatc	7775	
ngfb		Neuropathy, hereditary sensory and autonomic, type V	4803	ngf	7808	
nos3	Alz	Alzheimer disease, late-onset, Coronary spasms, Hypertension, pregnancy-induced Hypertension, Placental abruption	4846	enos	7876	
notch2			4853		7882	
nox1			27035		7889	
nr3c1		Cortisol resistance	2908	gr	7978	
nrf1			4899		7996	
nrg1	Schizo	Schizophrenia	3084	hrg	7997	

				ndf		
nsf	Schizo	Panic disorder<br>Schizophrenia	4905		2228	
p62			2965 - gtf2h1	p62 is an alias for gtf2h1	4655	
ogt			8473	o-glcnac	8127	
p120			1500 - ctnnd1	p120 is an alias for ctnnd1	2515	
p85			5295 – pik3r1<br>5296 – pik3r2		8979<br>8980	
parp1			142	adprt	270	
pax2		Optic nerve coloboma with renal disease Renal hypoplasia, isolated	5076		8616	
pc		Pyruvate carboxylase deficiency	5091		8636	
pdzk1			5174	cap70	8821	
pkd			5587 - prkd1	pkd is an alias for prkd1	9407	
plat		Plasminogen activator deficiency	5327		9051	
plod1		Ehlers-Danlos syndrome, type VI<br>Nevo syndomoe	5351	lh	9081	
plscr1			5359		9092	
pml		Leukemia, acute promyelocytic, PML/RARA type	5371		9113	
pp1			5540 – ppyr1	pp1 is an alias for ppyr1	9329	
pp2a			5524 – ppp2r4	pp2a is an alias for ppp2r4	9308	
ppara		Hyperapobetalipoproteinemia, susceptibility to	5465	hppar<br>ppar	9232	
ppp1r15a			23645	gadd34	14375	
prb2			440083	ps	9338	
prdx3			10935	aop-1	9354	
prkcabp			9463 – pick1	prkcabp is an alias for pick1	9394	
psen1	Alz	Alzheimer disease, type 3<br>Dementia, frontotemporal<br>Pick disease	5663	ps1	9508	
psen2	Alz	Alzheimer disease-4	5664	ps2<br>pre2	9509	
ptk2			5747	fak	9611	
ptpn11		Leopard syndrome<br>Leukemia, juvenile myelomonocytic	5781	shp2<br>sh-ptp2	9644	

		Noonan syndrome 1				
pxn			5829	paxillin	9718	
pygm		McArdle disease	5837	glycogen phosphorylase	9726	
qrsl1			55278	gata	21020	
rab38			23682		9776	
rab3a			5864		9777	
rad9a			5883	rad9	9827	
rai			shc3 - 53358		18181	
ranbp2			5903		9848	
trp-2			1638 - DCT	tyrosine related protein 2	2709	
rapsn		Myasthenic syndrome, congenital, associated with acetylcholine receptor deficiency	5913	rapsyn	9863	
rb			5925 – rb1	rb alias for rb1	9884	
rbl1			5933	p107	9893	
rela			5970		9955	
reln	Schizo	Lissencephaly syndrome, Norman-Roberts type	5649	reelin	9957	
rgs4	Schizo		5999		10000	
rin1			9610		18749	
ring1			6015		10018	
rpa1			6117	rpa	10289	
nov			4856		7885	
rpl22			6146	eap	6146	
rrm2			6241	r2	10452	
rybp			23429	yeaf1 – Entrez	10480	
sap18			10284		10530	
sap30			8819		10532	
scap1			8631	skap55	15605	
sgk			6446		10810	
sh3glb1			51100	bif-1	10833	
shb			6461		10838	
shc1			6464	shc	10840	
shp-1			5777 – ptpn6	shp-1 is an alias for ptpn6	9658	
six2			10736		10888	
six4			51804		10890	
slc2a4		Diabetes mellitus, noninsulin-dependent	6517	glut4	11009	
smad1		Charcot-Marie-Tooth disease,	4086 – smad1	madr1	6767	

		type 2D<br>Spinal muscular atrophy, distal, type V	2617 – gars	smad1 is an alias for gars	4162	
smad2			4087		6768	
smad3			4088		6769	
smad4		Juvenile polyposis/hereditary hemorrhagic telangiectasia syndrome<br>Pancreatic cancer	4089		6770	
smox			54498	pao	15862	
sos1		Fibromatosis, gingival	6654	hgf	11187	
sox10		Waardenburg-Shah syndrome<br>Yemenite deaf-blind hypopigmentation syndrome	6663		11190	
sox2		Anophthalmia 3	6657		11195	
sp1			6667 - sp1		11205	
sr			6344-sctr<br>6623 - sncg	sr alias for sctr, sncg	10608<br>11141	
scap			22937 - scap		03300	
srf			6722		11291	
stat5			6776 – stat5a<br>6777 – stat5b		11366<br>11367	
syk			6850		11491	
syn2	Schizo	Schizophrenia	6854		11495	
taf4			6874	tafii130	11537	
tbk1			29110	nak	11584	
tr		Pituatary adenoma<br>Thyroid hormone resistance	7067 – thra<br>7076 - thrb		11796<br>11799	
tgfb2			7042		11768	
tmpo			7112	tp	11875	
tp53		Adrenal cortical carcinoma<br>Breast cancer<br>Colorectal cancer<br>Hepatocellular carcinoma<br>Histiocytoma<br>Li-Fraumeni syndrome<br>Multiple malignancy syndrome<br>Nasopharyngeal carcinoma<br>Osteosarcoma<br>Pancreatic cancer	7157	p53	11998	

		Thyroid carcinoma				
tp73		Neuroblastoma	7161	p73	12003	
tradd			8717		12030	
trrap			8295		12347	
tyrp1		Albinism, brown	7306		12450	
vcam1			7412		12663	
vcl			7414		12665	
vegf		Diabetic retinopathy, NIDDM-related, susceptibility to	7422		12680	
vws		Orofacial cleft 6<br>Popliteal pterygium syndrome<br>van der Woude syndrome	7452 - vws<br>3664 – irf6	lps<br>vws is an alias for irf6	12728	
wnt1			7471		12774	
x123			9413 – c9orf61	x123 is an alias for c9orf61	24820	
xbp1	Bipolar	Bipolar disorder	7494 – xbp1<br>7495 – xbpp1	xbp1 is an alias for xbpp1 and xbp2 is an old name	12801	
xpo1			7514	crm1<br>exportin1	12825	
yy1			7528		12856	

** the association to the autism was taken from the following article: Wassink TH, Piven J, Vieland VJ, Jenkins L, Frantz R, Bartlett CW, Goedken R, Childress D, Spence MA, Smith M, Sheffield VC. Evaluation of the chromosome 2q37.3 gene CENTG2 as an autism susceptibility gene. *Am J Med Genet B Neuropsychiatr Genet*. 136(1):36-44. 2005.

D.2 – List of Arcs

D.2.1 This table lists all of the arcs that exist on the graph. For each arc there is a list of the PubMed IDs of the articles from which this connection was extracted.

ARC	PUBMED IDs THAT SUPPORT THE ARC
abl1->eif4e	10753870
abl1->pxn	10593973|9202056|7534286|7493940|8631923|9603926|11864995|10704446|10970852|12861019|10436023|10825157
abl1->tp53	11486026|9037071|10085066|10629029|11960694|10391249|12594211|8900110|10391251|10805805|10611241|11847100|12021410|10347217|9890940|10753870|11375889|11323398|9628871|10202145|12215537|10082540|9891054|12620407|10882116|11376656|12620409|9883720|11889036|10574931|10625600|10866655|10436023
aes->creb1	10660609
ahr->arnt	10688617|9271342|9111057|12058065|11179451|8940186|9211913|10722677|10913191|9447995|11046109|11093792|10871357|9547360|11752201|9765510|8647831|7592839|9079689|9020169
ahr->egf	12606443
akap5->g72	9915845
ampk->cftr	10862786|12427743
ang->jun	10864918|9169474|10325245|10764405|12770924|9756884|10764657|9758646|11282899|12054679|10087056|8939945|11994255|8978332|9564040
ap-2->egfr	7534311|11133812|9242916|8548289|9950686|8999911
ap3->centg2	12967569
ap3->hspg2	9188501
apaf1->bcl2l1	10449754|9539746|9488720|9829980|11961058|10791976|9878060|9651578
apbb1->app	11283011|10075692|11517218|9837937|11425871|10373567|11337355|12843239|9045663|9553108|9685356|10702315|11544248|11907044|10557205|11441186|11517249|11606623|12196555|8537337|12084708|11959904|11119687
app->psen2	11847232|11756438|10026204|10823929|10617607|9573389
app->tp53	12609999|10377452|8833912
arnt->psen2	12754377
atf3->creb1	11792711
atf3->tp53	11792711|12161427
atf5->ngfb	12805299
atp8a2->blmh	11976340
atr->chek1	10859163|11535615|12672690|12791985|11700302|11953432|12147700|12399544|12554670|11799063|12529385|11390642|11790307|11278490|11553781|10973490|12629044|10859164|11313465|11090622|12554671|11481475|12526805|12620223|11544175|12660173
baat->ppara	10438514
bace1->app	11740561|12458195|10531052|10924510|12480937|12645527|11742091|12470796|11055423|12670422|11141490|11741910|11071887|12878199|12087104|11832221|10931940|10591213|12801932|11316808|12471021|11423558|11466313|11698669|12515826|12901838

bad->bcl2l1	8626425|11572856|12176160|10949026|12522090|11455016|10617621|12464623|11278671|10455159|10330186|10595916|9305851|9388232|10383455|10903316|11020382|12084714|10725400|8929527|11884367|10579998|11287608|12351720|12629175|12453482|12019309|7499206|8663437|8995241|9525930|9660836|9685326|10066754|10358075|9736715|9636168|8929531|11121444|10769035|10747099|11032809|10202146|10330169|10195903|9812896|9721091|9381178|12676358|11259767|12363409|12711631|11007881|11312610|11839804|10521512|12657644|11159738|11104747|12119196|12727206|10811615|11839824|11259582|11463831|11027273|12782668|11359940|10679322|10884318|9581757|12215543|9507158|10551876|10788521|11717309|11983683|9346240|12729578|9716401|12808093|9726976|10574959|10837473|10837486|11278822|11790791|11493700|9770514|10950869|12944463|11784871|11154272|10594016|7552172|11485901|11723239|10692444|11157722
bad->sos1	11571297
bak1->bax	11571294|12454021|11340162|12689595|11447222|12368257|11402069|12167710
bard1->brca1	12732733|10764811|10859161|11925436|12431996|9832560|11320250|9342365|11278247|11904382|10026184|11927591|12832489|10741968
bard1->tp53	11779501
bax->bcl2	11971968|9584191|8621473|9708813|8943258|9556634|11279112|12642586|10980124|12401491|9482724|9202007|9395483|9525867|10383455|12702731|12729583|9303307|9396780|9096388|11226327|12433363|11158585|9361016|10639111|10487825|11395384|9096145|9437022|8816704|11489446|10217402|9553144|10085086|9888861|8929527|9445173|12535734|10747022|10024594|9395403|9219694|11080152|9826712|10576740|10100604|12379660|12215543|11470757|11082422|9111042|10725400|10479647|12021051|9916015|9934848|8840837|9054761|9639374|11094032|9721694|11166763|9171341|10669738|9891071|8647929|9708817|10702418|10934149|11368946|9974117|10677604|9379849|11687278|9454852|9660917|10867216|8755480|9460752|10391451|9497329|10753914|10085289|10359739|10825507|12828932|12130671|9581757|10970901|9507158|8663032|9382873|8631771|9315550|11124420|11805214|12466137|9812996|9463381|9763432
bccip->cdk2	10878006
bche->atp8a2	12181345|12192057|9238019|12039045|10652347|9722535|10831595|9843509|11997133|12965201|10381780|8798754|12370317|11035801
bche->e2	10788482|12218038|11013251|12239214|12626763|11057907|11323428|11092882|9539738|12574131|7822313|8617766|9727038|11304544|9892642|11301008|11893341|7890655|7896852|8537366|11285267|11739378|12756305|11752427|9843509|11889054|10856250|12370317|11781147|11259571|8576261|10882734|9611220|10934274|7721865|10648794|11744158|9374509|12207029|9374507|9804845|9915800|9933649|10652347|10966472|12192057|10594006|10559424|7592989
bche->e3	12626763|9727038|8969175|11950839
bcl2->psen1	10521466
bcl2l1->bax	11150501|9927423|9182591|11278245|11861645|10330404|10669738|10629050|10490606|9556634|12591950|10617621|11413128|11852059|9989610|11782444|8798452|12767928|10753914|12642586|10737788|10318911|10085289|9463381|12368257|9742125|9742122|14499110|12351720|12629175|12540825|11402069|10228148|12965200|12419244|10757797|11094149|9111042|10872455|8626425|10884318|12853473|9581757|11278320|11967212|9395483|9488720|9890985|12783855|10444588|11604501|10400666|10446202|12095614|9020082|9346936|10446169|9726976
bcl2l1->psen1	10446169
bcl2l1->psen2	10446169
blr1->lta	12354388
bmp2->smad1	11408477|12923053|12586333|9006934|10473586|11755539|9442019|12493742|10903423|11395480|10224145|11700304|9748228|12060751|10232991|11038252|12514223|12589053|12535648|9436979|8653785|12370242

bmp4->vegf	12235106
bnip3->bax	10891486|9575197
bnip3->f2	10891486|12169648
brca1->sp1	12706836
brf1->mapk1	12743036
brf1->tp53	12773395
calm1->camk2g	10377038|9790528|11853966|12764111|12788814|12488453|8626500|11919178|8749377|10777771|11561078|9768845|12531901|10462051|9620694|8702694|10473573|12414535|10575025|11408552|8636075|9422695|10858498|9694809|10722875|9858538
calm1->rgs4	10747990|11463389
camk2g->nsf	11278345
cap1->calm1	10770930
cas->pxn	12065577|10712510
cas1->il18	11953408
casp3->psen2	9353287|10551803|10069390|10446169|9990034|10409650
casp8->psen1	10069390
casp8->psen2	10069390
cav1->app	9553108|10414982|10523618
cav3->app	10414982|10523618
caveolin->apoer2	11369809
cbl->akt1	9507028
cbl->bcr	8621719|9020138
cbl->egfr	12604776|11514635|9079708|11053437|7782294|10521482|12070153|12734385|11408594|11071924|11853876|7657591|10531381|11406619|12631709|11223028|10635327|12593795|10788520|11823423|11741535|11223029|10490623|9851973|9710588|10617627|8798643|9525870|9593726|10428778|9653117|9653112|9759494|11864992|11756476|10769031|10637223|10880467|12697834|12024036|10629042|9858595|11179425|10888670|11029044|12479806|10882103|8662998|12649282|9663390|11713248|10490604|10608804|11375397|10571044|11562344|10816576|9130710|11448952|11756475|11971965|11950941|12842080|12890531|11983899|10369675|12411582|11551499|11071886|12239347
cblc->syk	9535867
ccl4->sos1	12482598
ccnd1->tr	12048199
ccne2->cdk2	11687642|9858585
ccs->il10	11292741
ccs->jun	11532864
cd160->sos1	9488442|8662798
cd28->pp2a	11021529
cdk2->sp1	12794070|11598016
cdk2ap1->cdk2	10938106
cdk5->mapt	11305318|11675481|10526177|10878257|7704571|9200503|9878757|8795628|10460255|11124971|11160434|12533609|12598607|12684440|12832520|11583627|12600719|11445251|11343650|12765608|9219970|12207971|11065131|8892394|11520930|

<table>
<tr><td></td><td>11121021|8662984|9038181|11812770|12536148|9614189|10604460|11086171|12387894|12604663|10571240|12062036|12372285|12435421|10749861|11443123|10706614|12151531|10884378|10500146|10982801</td></tr>
<tr><td>cdkn2a->mdm2</td><td>9724636|12086884|12860999|11416144|11909952|10801444|11223031|12297306|11099028|11485924|11359905|11739734|11507088|9529249|10713175|11278317|11909522|10541552|9765200|11106551|10360174|11877395|10593882|11748239|11867628|12524457|9653180|11532955|11731475|10995391|12944468|12582232|11223036|12531896|12370303|12486224|11274364|11839803|11438662|11960694|12049738|12887889|11382768|11518711|12446718|11839577|11297540|11459832|12842086|11971980|12529318|11331603|11960918|10753221|10334193|10338215|10199402|9529248|12099684|12200117|12589812|12426395|11250899|10205165|10202144|10357817|9878046|12628936|12435633|12464630|11390353|11114893|10898794|10541553|10485844|9851971|9832500|9765202|9694791|9694806|9694807|12640134|12640129|11756559|11739748|11739724|12077343|12101227|12417717|11238948|11564860|11113205|10688649|10648628|11003658|10891494|10594030|10207115|9632782|9774662|9742086|10436023|9651526|12086853|12150825|12242155|11752120|9774968|11511364|10911993|10983977|12154087|12151395|12493762|12167711</td></tr>
<tr><td>ced-4->bcl2l1</td><td>9742122|9581757|10846174|9382837|9027313|9463381</td></tr>
<tr><td>cfdp1->eif4e</td><td>9049310|10654941</td></tr>
<tr><td>cftr->atp8a2</td><td>10788430|7493947|9325282|9346969|9463368|10581363|9593757|10518014|8617738|9065435|9813087|7530246|8910333|7545672|10660517|11112782|10581360|7730361|11597353|12225848|8910473|8617745|10488089|11904291|9640644|11262417|10880569|8662751|11932230|9064787|9268333|9724814|10675364|10764752|11110786|11937500|9482946|12948585</td></tr>
<tr><td>cftr->vws</td><td>11254634|11997458|11179370</td></tr>
<tr><td>chd3->hdac1</td><td>9804427</td></tr>
<tr><td>chd3->hdac2</td><td>9804427</td></tr>
<tr><td>chd4->hdac1</td><td>9804427</td></tr>
<tr><td>chd4->hdac2</td><td>9804427</td></tr>
<tr><td>chek1->g72</td><td>10330167</td></tr>
<tr><td>chrd->bmp4</td><td>9689081|10354471|10938274|7552158|11260715|10490096|9827801|9458049|9363950|9363949|9182762|9118218|9118219|9019398|8752215|8752213|10085302|9885250|9660882|9545242|9427753|9233797|11179208|11483516|10733523|10601040|9716398|9620855|9585504|9367978|12897139|10688656|9710639|9854046|11298453|11260717|12618130|11782317|12062089|12586065|11493559|11526070|11493569|11532920|11532917|11566848|11546754|11585808|11714670|11731450|11807034|12070085|12397106|12403713|12668635|12736214|12835400|12835401|12874126|11992721|9545539|9651472|9651485|9831647|10096059|10096060|10415349|10415355|10446267|10446269|10495269|10495268|10559482|10559480|10642437|10727860|10704847|10781939|10842063|10960783|11287189|11404077|11472837|11850177|12351167|12464431|9675418|9324353|9278524|9547239|10492013|9660951|11336702|10518210|12727446|11182081|11856527|9832510|12629212|9056707|12702725|8895454|9182796|9697853|11343645|11830566</td></tr>
<tr><td>cks1b->cdk2</td><td>12007796|11349131|8601310|11463388</td></tr>
<tr><td>clathrin->ap-2</td><td>12429842|10944104|9571248|12589051|12086608|9817753|12832620|10748223|9724631|11451993|12086597|12847086|10428863|10770944|10692452|11756481|10801423|11382783|9384576|10022830|9495551|9171339|10811610|8662627|10364223|11042179|11266448</td></tr>
<tr><td>clk1->eif4e</td><td>10648556</td></tr>
<tr><td>cls->cftr</td><td>11118444</td></tr>
<tr><td>cls->egf</td><td>12829236</td></tr>
<tr><td>coh1->fn1</td><td>12010974</td></tr>
</table>

creb1->jun	10779351|9447994|12721099|12650978|9038229|11136722|10825388|8662742|9668114|10318823|7554179|12433834|9155027|11416131|12909347|11504868|10719210|10871295|9668047|8662977|11976688|11438531|9819380|12676525
creb1->sp1	11870072
crk->pxn	9722546|10825157|11884618|12857867|8631760|10329689|10976102|10704446|10385525|12515828|12475948|9888877|11432821|8662907|7534286|11752449|9756887
crk->vegf	12138161|11956190
crkl->bcr	8810278|10608804|12124177
cul1->myc	12769843|12769844
dag1->pkd	9507012|11410587|11729268|9295346|9607313|10856238|11786634
dapk3->daxx	12917339
daxx->tp53	12954772|12482984
dcn->egfr	10913155|9988678|10942781|12105206|10383378|12900463|8702560|8702652|8702651|8943211|9079625|9115237|9452417|11071883|12135914|10684260|10459005|9024701|10747004|9202067|12871697|9553109|11152695|10077642|10872465|12695504|9878054|8895468|11406617|12621051
dlg1->akap5	11943807|10939335
dp->apc	9152393|9694973|12649388
e-cadherin->psen1	11226248|11953314
e2->eralpha	11279159|12135905|10967123|10681512|11369763|11700319|11751902|12411396|12612060|12897156|11971969|11493692|10491630|12482819|11751931|12376702|12021387|9765300|10805720|10542244|11356845|11481323|11555647|11823467|12547825|12682072|9716542|11452016|12588759|10982795|12821541|12588983|10611355|10727250|11160157|10611082|11502892|12566449
e2f1->bax	9774967|11739724|12149244
e2f1->sp1	8647824|9160753|12220517|12788094|9694791|11259576|11564866|9584162|9819401|10436023|10521653|11992719|11433027|12364593|12799438|12888513|12077144|10514422|10187822
e2f4->ccnd1	10611246|9584162
e2f5->e2f1	10823896|12177046
edf1->calm1	10816571|9795107
egf->eif4e	9829969|10859165
egf->jun	10491655|12621033|9169474|10611224|9342344|10197981|11756554|12069834|12459251|11223155|11463380|7629150|8940130|9099730|10753873|10652359|11134003|11371563|11592962|12446727|11416221|9419345|9817764|10802070|10779323|9528756
egf->sp1	9195964|7890769|10973489|9162042|9169507
egfr->mapk1	11701462|9648731|11809859|10325245|9685397|10734107|10851244|10644690|11319218|12657625|10677489|12119356|11003669|9710602|10888683|11341977|12124174|12435806|11861812|10523301|10411909|11592962|12426305|12242303|12016209|10777553|12421825|12024020|12897150|11889128|12391274|12135609|12869389|10490623|11788593|11046132|12058069|12438509|12522117|12654926|11160627|12101233|10567720|9632734|11278472|12459458|10419448
egfr->rgs4	11602604
egln1->cycs	11060309
egr1->psen2	10982796
egr1->tp53	11035041|9242687|12629205|9368051

eif4e->ccnd1	7673150|9724638|9218810|11500381|10825184|10454551|9891068|9447982|10960064|11160419|10811643|10567390
eif4g2->eif4e	10409745
emx2->wnt1	12668639
ep300->tp53	10779327|11591713|10487758|9891062|9891054|11511360|10518217|9830059|10438470|10777477|10779504|10625690|11013253|11076933|11279157|11073948|12068014|12493762|12514180|12499368|12501250|11070080|9789016|9759497|11323398|11779456|11672523|11250899|11707411|10887150|9744860|9649502|12773566|11564863|11486036|10648616|11046145|10982847|10891493|10454583|10207073|10022902|11701890|11099047|11058129|9809067|9885574|12426395|10629029|11782467|10942770|11036071|9215639|10318779|10567378|10625687|11278389|11382768|11438528|12609999|11223031|9342368|11158315|12040123|12690203|10485844|9890940|10906119|10570149|10359817|9501176|9724739|9636169|9618472|9600977|11960904|10025405|11672522|9430646|10202144|10202145|9878046|10673500|10673501|9765200|9407038|11713287|10710310|12582232|11259528|11486030|9528808|9575138|9724748|8945521|8616895|11742995|11073993|11511361|10480893|10733570|10547702|10891494|10207094|12718889|9407140
eps15->egf	9920862|11423532
eps15->egfr	10953014|9950686|11461694|9490719|9813051|7797522|9694904
eralpha->xbp1	12954762
erbb3->nrg1	9756944|7673253|8617810|11555649|9312020|12204533|9049301|10970856|11867753|9275162|12297049|7642563|9694850|8653786|12297041|9566971|12727221|10521398|12052879|10101228|11337203|10399934|10707974|10839362|11498047|9593726|8631797|9786961|12788100|9774339|10369675|9030624|11027222|12556529|10725395
erbb3->rgs4	12840049
ern1->atf6	10856300|11821395|11779464
exo1->ap3	12738886
fadd->casp8	9915859|10938285|12063258|10849436|11486001|11094078|10454539|12529427|12556488|12167698|11279061|12477721|11739735|9832562|10409763|12130683|10744735|12556501|11784850|10026132|9054728|10490590|9606216|9695946|9215629|8681377|9311998|10187805|9742122|9488717|10208979|10926929|9228018|10894160|9553074|10934188|11729181|12220669|11346652|11514566|11577081|9990010|12881424|11703941|12354390|12507932|10493725|11230124|12354397|11395500|12702723|10832033|11485733|11600497|10611305|10579724|12753742|9257699|9144192|8939562|9268381|10593992|10823821|9037025|11384975|10966458|12191486|10753878|11821416|12702765|12646168|9740801|10224298|10383428|11016920|11085984|11427719|11420043|10318846|11278725|11606776|10952991|10988287|11114298|11461927|11581255|12482880|12482751|11717445|10411938|11756235|9606215|9501089|12198154|12832037|10894161|12620239|10542291|10688869|12401196
fas->casp8	12021072|11330312|12354397|9694885|10849436|11035014|11121439|10769034|9884343|11861801|10611959|12670865|12167698|10958671|10594023|12069853|11369777|9832562|12556501|11410528|10891503|11136242|10403638|12529427|10619428|9891071|10412980|9606215|10952962|12031968|9774341|12556488|12101236|11359904|10805722|9931493|10623660|10381555|9729047|10366635|11549719|11713262|11279218|12887914|9341131|9753321|7536190|7642544|8663439|8617712|8617770|9452459|10318846|11733517|10393932|9108051|10966458|9727492|9727491|9184224|12529384|9027313|9054009|9027312|12840065|10837247|12499380|10187771|10419518|10744730|11320092|11514566|12145284|10860997|11980919|10579724|11179211|9556450|11389967|11337381|11311984|10230394|11067865|11919192|10506188|10521451|10747891|10801801|11447169|9468507|10940305|12668660|11566253|11420043|11384965|10662780|10617566|12917351|10832033|11420045|12019321|10619023|10949027|12097334|11925448|11278459|12832037|10218585|9516458|11679590|10411938|10194469|11696559|11500358|10364155|11978827|10751382|12354390|12097332|11717445
fes->bcr	8955135|10567558|9218495|8663427

flj10261->nsf	9728906
fn1->bcr	11566866
foxj1->cftr	9096351
frs2->grb2	9182757|10092678|10629055|11447289|11997436|10748052|9268386|11562460|9880324|12181353|11911873|9809073|10748077|12419216|11353842|10330161|9632781|12606343
fut1->il10	9417080|11564441
fyn->mapt	11546790|11826099
fyn->vws	9294206
gab1->egfr	11134009|11896055|10648629|10551845|11916960|12024020|12686619
gab2->bcr	12124177|11278704
gab2->egf	10973965|12464621
glur2->grm3	11549753|9697854|12700243|12062022|11595203
glur2->nsf	9728920|10595516|12011465|11416187|12052905|11119696|12441055|9697855|11348590|9697854|12062022
grap2->grb2	9857184
grb2->bcr	11726515|12124177|8995449
grin2b->camk2g	10764765|9694809|10077668|12650977|10827168|11222640|11530236|11459059
grip1->glur2	12458226|11007883|10896157|10436050|11931740
grp->atf6	9837962
gsk-3->mapt	12937146|12765608|12551948|11812770|11119717|7704571|10377336|10899437|10878257|11278002|11943212|11959122|10455163|12095675|11438574|10938436|12069822|12177184|9235959|9312151|10601262|11861647|9635432|12527198|11943712|10700568|10662688|11033082|9771888|11094148|11086171|11257498|11696357|12387894|11083918|12062036|10465442|12435421|11602330|10899427|12147701
gtf2i->atf6	11287625
gtf2ird1->gtf2i	11713284|12475981
hbp1->rb	10702291|9671483|11486012|10562551
hbp1->wnt1	11500377
hck->bcr	10749872
hd->sp1	11988536|11839795
hdac1->tp53	12672825|10777477|12426395|12665570|11994312|12068014|12697811|12408818|11779456|11672522|12556448
hdac2->sp1	11311229|12176973
hdac9->creb1	10428804
hdac9->pml	11259576|12954772
hdac9->sp1	12176973|12788094
hdac9->tr	10921888|10734189
hgs->egf	11003664|12686592|11916981
hgs->sos1	12686592
hipk2->pml	12045187|12954772
hipk2->tp73	12954772
hlf->creb1	10364202
hlf->gc	10848603

hlf->il8	11895948
hlf->vws	9453600|9079716|11083760|10456914|10708745
hps1->ap3	12663659
hrmt1l2->yy1	12704081
hspa5->atf6	12110171|12853141|12727195|12808051
hspa5->atp8a2	9748217|9202129|11943137|10428866|10367885|8702846|9020152|9030582|10816561|9199165|9843586|11485742|7737982|7629200|9006964|10893409|12411443|7649989|9305856|9038234|10611978|8626673|10400622|12446677|7730335|9545266|10777498|10613903
hspa8->atp8a2	8824236|10318904|11683998|7592893|8702492|8702942|10075921|11909948|9774640|11222862|10397752|9312080|9545310|10454533|10938100|10625686|11854498|12040123|11395008|10809723|9006893|12426384
hspa8->cftr	10982807|12039948|9325249|10075921
hspa8->tp53	9235949|11707401|8663025|10075665|8940078
hspd1->casp3	12729585|10205159|11282911|11230124
icos->nfatc1	12818161
igbp1->g72	11371618
igf1->nrg1	10601306
igfbp1->igf1	9325295|9446566|9733769|11546760|8958208|10407151|10704627|11231911|12119297|8921789|11063745|11834727|12163461
il18->tp53	11470273
il18->vws	10101231|11500393|11056157|9449707|10816504
il5->ccnd1	11498591
irf->sp1	10364249
irf->vws	12821121
jun->ap3	11526073|11959818|11950941|11226177|11832239|12271125|11033084|12486136|11707398|11071902
jun->sp1	11110778|9092567|10838085|12633873|8647847|10364249|12606768|12093805
jun->vws	12133836|8690807|12750010|11842088|12003967|10848596|11959104|10768925
kcna4->fyn	11149959
kl->il10	7531701|8703029
krt14->plat	10449779
lef1->ccnd1	10318916|12183361|12571228
lef1->creb1	9584163
lrp->app	10899157|10940295|11823454|11067868|10550330|12417655|9582384|12547833|10702315|9837937|11245926|12117549|10218790|12411487|11907044|11606623|9478949
lta->tp53	11867746|12665584|12150956|11156605|11884599|12676586|12232053|10544235
lu->egf	10556937|11238942
maea->fn1	11698365
mapk1->myc	11018017|10551811|8663331|10037749|11408569|7535770|9111004
mapk8->mapk8ip1	11790767|9235893|11378392|10748095|10490659|11562351|11432832|12433842|12511654|12524447|12194869
mapk8->myc	10551811|11567045
mapk8ip1->apoer2	10827173|10827199|11238452

<table>
<tr><td>mapt->apoe</td><td>9272834|11988340|12383924</td></tr>
<tr><td>mapt->psen1</td><td>10341227|9689133|11157069</td></tr>
<tr><td>max->myc</td><td>12408820|9312026|11511535|11602341|10744738|10684809|9150133|12553908|9451002|9835654|9184233|10811619|10601024|10508165|12695332|10465786|9887096|11447169|11154258|11134351|11073985|10825189|10207076|9528762|8601634|9062341|7583141|9197243|9714754|10333519|11111045|11937633|9115440|9634703|9491997|10078200|11030351|7499403|8075979|12080043|10899308|12486219|12747840|9804832|12207913|12637567|10781542|10205150|11532874|8081751|11891322|9211884|9506959|9856992|12620195|9632747|9653143|10835425</td></tr>
<tr><td>mdc1->brca1</td><td>12611903</td></tr>
<tr><td>mdm2->psen2</td><td>12667452</td></tr>
<tr><td>mdm2->sp1</td><td>11226218|12702724|9399940</td></tr>
<tr><td>mdm2->tp53</td><td>12944468|11852039|9858609|8875929|11278372|12068014|10688868|12426395|11013216|11511360|11507088|9419352|10202144|11094089|10085066|10488081|10722742|10777561|10827196|10884389|11572869|11821415|11948180|11923280|12244051|11960904|9878046|11331603|12832478|12167711|12370303|11713287|10757806|10648610|10629057|11046142|9819415|9742086|11960694|12932324|11223035|11223036|12586367|10075719|10781582|11016968|9653180|9759494|9694806|9649502|10688649|12124178|12753897|11297540|11470783|11591713|12860999|10710310|12297306|12646216|10436023|9271120|10075736|9363941|11250899|10027414|12604636|10205143|11387205|10357817|11711428|9694807|10207094|12040123|11125034|9724739|10744705|10347217|10788439|11340074|11397792|11053443|12821780|11395416|10892746|12507430|12526791|11850407|12612087|12077343|11713288|11486026|10713175|11397945|11397937|9450543|12832479|12529318|10747924|11323398|7953514|12519780|10900010|9430646|11839577|12842086|9182591|11279110|12407176|10077639|12807744|12927808|11562347|12897156|11739734|12101245|11359905|11027272|10913191|12867035|12620409|11178871|10688665|11431470|12082016|11836256|12101227|11152666|11880381|12606552|11070080|10075936|12204530|9413984|10906133|12493762|11805282|9576951|11847101|10594030|9418898|11755215|12718885|10716710|11781307|11532955|10449408|12640129|10747932|10747903|10791951|11013229|11821411|11877395|9529248|9529249|11909522|12890487|10562557|10562558|12628936|9832500|9765200|11238917|11238948|12242109|10393843|10887150|10660586|12527807|12232053|12424231|9418889|9600935|11113205|10373523|10330179|9858527|12642583|9721090|10980197|11057904|10541552|10218570|9182520|9278461|11867628|11172041|10874024|12509446|9685342|9830059|10318779|9890940|10993878|11568180|11714701|11729185|11799106|11504915|10097151|10611293|10570149|10359817|9501176|9636169|9618472|9600977|10966458|11960905|12082025|12189181|12376469|12376468|11375889|11323386|11323395|11285191|11181441|11751435|10753221|10753197|10334193|10545420|10338216|10338215|10199402|9215639|9039259|8756720|11672523|11030628|12654245|10366599|12099684|12200117|12589812|12646252|12604359|12659830|12821135|12943690|9564034|9724636|9707426|9606193|9034333|9312058|9250682|12356735|11179235|10205165|10202145|10064595|10601022|10581258|10523305|11782444|11159909|11390353|10673500|10673501|11114893|11018017|10995391|10970882|10950866|10898794|10215620|10541554|10541553|9925639|10521394|10485844|9472015|9851971|9765202|9744860|9732264|9694791|9679063|9407038|12242235|12529408|12897130|11865061|11839803|11756558|11756559|11739748|11739724|11909952|11909939|11971980|12077346|12138209|12192050|11158294|11158315|11564862|11564860|11438675|11416144|11313463|10733583|10733572|10648616|11003658|11003642|10891493|10891494|10779331|10779327|10373532|10207115|10082540|10082548|10022862|9891054|9632782|9632751|9774662|9733514|9421473|12084459|10771089|10978505|7583121|10801444|11696333|11818069|12050146|12810584|10721718|9651526|11335127|10982879|11812854|12582232|9736038|10595915|10793069|10793065|10854221|11021837|11238062|11943705|12414510|11457508|12086853|12150820|12150825|12242155|11259528|12235242|9774968|9885574|10078201|10360174|11239457|11511362|11511364|11511361|11741533|11779495|11779500|10911993|9927611|11562441|12644594|9808466|12552135|9323137|7649977|7592643|9388200|10438519|10608892|11359766|11080152|11909962|12138191|12711691|12781359|9668066|11</td></tr>
</table>

	035041|11953432|12372299|10391892|9890939|10593882|10930428|11278754|11076933|11259404|11384992|11756437|1195 3423|12145279|12154087|12488444|12538596|12475992|11818530|12172011|11309506|11158620|11070090|11035798|10860 994|9861017|11283316|11062168|8945521|8674115|8616895|11672522|12419241|12147236|11532927|10970867|10487758|11 731475|10673502|10887155|9887100|12640139|12101228|12417717|11486030|10733570|11073966|10207112|10403253|9382 809|10521653|11485924|11720709|12620407|11226425|11223031|12049738|12408818|12667452|11860280|8662825|1058873 7|10562283|12411495|11459832|12167722|10779353|9891077|9858587|9733515|9792667|11867746|12408820|11309410|9488 468|10906337|8674108|9742130|11048728|10606744|10801797|10958792|11283254|10781812|9786925|10329737|10734067|1 1707453|11847229|12091386|12427754|12514180|11983915|11158615|9482877|10872455|12411481|11285227|11544175|115 28457|10381566|10702413|11395384|12086842|10567715|12593856|12697828|11359902|10454584|10523633|11937641|1110 6551|9774967|12690203|10358050|11279015|11172000|11756220|9741624|9351830|9321401|11707411|10640274|9832503|95 85502|12101234|12446780|10825186|9488478|9488444|9742084|11997093|9368757|9443911|12477391|12718877|10518217|1 2606767|11196623|7534296|7890669|8910602|9417035|10446138|10506209|10660629|11278685|11342538|11375995|125197 69|12397175|9751767|10712385|10764661|12584171|10791968|12665584|11940651|11604501|10913196|11102515|9716124|1 2729918|11879638|11328884|10922493|10856296|12925764|11704667
mef2d->egf	9448004
mitf->bcl2	12819038|12086670
mitf->tyrp1	12351177|12136092|11734563
miz1->ep300	12244100
miz1->myc	9312026|10866684|12110671|9927431|11602341
mkl1->srf	12732141|12944485|12397177
mlana->egf	11734563
mllt4->bcr	12808105
msx2->bmp4	9851982|10954731|10773441
myc->bcl2	11158311|10359817|12015982|9430646|9878046|12628936|10541554|10541552|10541553|10444588|10364155|9716401|9694 806|12167710|11438662|10913183|10779339|9020077|12718877|9584184|9735050|12149248|9360929|11604501|12086865
myc->pkd	12732727
myc->sp1	11274368|11804592
myc->tp53	12080043|7499359|12049739|10995749|8929532|11350949|10490652|9632811|9364064|9724637|10541553|9694807|1083815 7|11960695|9694806|11278333|11402027|11805123|12524457|12189181|12628936|10541552|9765200|9765203|11604501|127 11674|9677415|11923280|12408868|12450789|12408820|11431470|10688869|7614713|12909717|10364155|12086865|124905 73|11577010|11274368|12842909|11438662|10637230|10075936|12391152|10779327|10488335|10097151|11940651|9721090| 10347180|11821411|12538581|10962037|9466972|10619024|10102818
nab1->egr1	11042387|12379479|10514413|11111043|10364563|9418898
nab2->egr1	12379479|10514413|10079243|10364563|9722618|12556466
napa->nsf	12738684|9456319|9430666|10716927|10369670|9030619|11294904|11031229|8550603|12554740|9362506|10487746|109304 51|10954749|10075705|11301340|10831610
ncoa1->ccnd1	9832502
ncoa2->tr	11084025|9368056|11689447|9624051|10454563|9920895
nedd8->cul2	9268381|9353319|9556629|9452416|10075690|10207026|10585493|10722740|10828074|11024059|10097128|10318914|91221 64|12810083|10880460|10228155|9531529|9694792|9447969|10205047|10806345|11865071

nedd8->pml	10207026|9452416
nes->tp53	12944468|11397937|11847229|10075936
nes->xpo1	10991937|12519765|12563037|11425870|11486037|11489891|11551912|11781313|12374846|10611964|11149927|10075936|9679058|12167720|11604520|10454574|10490644|10801471|11287007|10215621|10779340|11743003|12193389|9808617|11073978
nf-kappab->sp1	10449442|10829018|9789016|10021463|11970949|11495920|10454561|9488441|11110778|10082535|10637333|12458222|9765204|8557630|10205175
nfatc1->vegf	12427739
ngfb->sp1	9236224|12606768|9712850|9506991
nos3->calm1	10683200|9325323|12228731|9410890|10455137|9188442|9856995|9287303|11701613|11090123|9765272
nos3->caveolin	12860247|11136695|8798458|9188442|9325323|9756862|9804784|9856995|9452418|11425855|10400908|9410890|10079111|10781589|12177436|11157661|10471811|11714864|11120737|9488658|11976335|11701613|10523618|11251080|12486234|10514497
notch2->jun	9528794
nov->egf	12050162
nox1->ang	11348997|11348993
nox1->vegf	11348997|11805326
nrf1->cycs	10777619
nrf1->sp1	8567667|12031478
nrg1->egf	7730382|9786961|9774339|9130710|9029149|9305636|9742126|10839362|7721889|8940074|8702681|9042959|8631797|10369675|10022883
nsf->atp8a2	7622514|9422733|9647644|8702750|10369670|11101518|9334216|12738684|9074632|9771883|9697855|10831610|12914953|8662851|9452464|12554740|10716927|11850454|8702539|9148916|9087439
ogt->ap3	9242909
ogt->sp1	12829252|9452489|10580136|12417416|11371615|12150998
ogt->tp53	11574421|10717000|12829252
p120->e-cadherin	10629228|9744888|12427869|12435727|9395537|11801604|11226248|10207085|10487836
p62->tp53	10506209
p85->cdc42	12566459|11038173|11278326|10212255|9150145|11057896
p85->egfr	8995250|11853876|11172806|11375397|10559227|12482824|11533253|7797556|9710588
p85->erbb3	7535767|9388271|9694850|10973965|11546794|9049301|8550620|10559227|11853876|11297548
parp1->yy1	9822623|9928945
pax2->egf	11262416
pax2->tp53	10980123|10908331
pc->prb2	10744734|10675519|12016218|9065419|9388264|9516463|9553075|10984480|10220410|11257523|10631296|11163216|10364092
pdzk1->cftr	11051556|11988170|11495894|10852925|11278980|11950846|12471024
pkd->syn2	10998417
plat->sp1	10842166|9665463|11477089
plod1->sos1	9716553

plod1->vegf	12606472|9371824
plscr1->abl1	11390389
pml->eif4e	10893273|12163469|10077561|11500381|10906119|12167712|12554669|12897141|11959093
pp1->ppp1r15a	11564868|12724406|12556489
pp2a->bad	12944463|11839804|12729918
pp2a->mapt	9221771|12904469|12937126|11086171|11749959|11441005|10681533|8982166|10464280|11121021|11520930|12937125|11473109|11997007|9744873|11781156|10700568|10899436|8621419|11904169|11090625|12435421
ppara->creb1	11943463
ppp1r15a->g72	12446838
prdx3->cycs	11285261
prkcabp->glur2	11891216|11466413|10027300|12597860|10985351|11931741|12052905|12011465|11007883|10399937
psen1->grm3	12700243
psen2->psen1	9050898|9452432|11738826|11090127|11904448|10652302|10518543|12198112|11741906
ptk2->egfr	12167618|11359909|12124174
ptpn11->egfr	12582165|11912208|10531446|11223155|10411909|10973965|11042184
ptpn11->shc1	9843570|10973965|12124172|11640895|11698646|9488729|11602579|9110989|11435425|9885561
pxn->igbp1	11598204|11517262|10781578|11477105|11533025|11825902|11919182|11134073|11121431|12695503|11784865|12808052|12221126|11359939|12729561|12871702|12445772
pygm->apc	8663301
qrsl1->vcam1	12493764|12547825|10884367|11590177
rab38->tyrp1	11917121
rab3a->apc	12426384
rab3a->bdnf	11517266
rab3a->calm1	11522668|12163174|11134008
rad9a->bcl2l1	11971963
rai->p85	12242309
ranbp2->cfdp1	9045717|9398662
rapsn->fyn	9136771|10400682|11356869
rb->mapk8	10799486|11157751|12297496
rb->tp53	9417035|9466762|10078201|10851232|9664074|12810584|9422723|10906133|11035041|9418885|11106551|9546379|10660629|10334193|10980197|11050087|12646568|12391156|10688869|11591730|9786955|9508781|12054599|9463382|9451002|9649429|11432828|12381663|11230146|10898794|9694791|9334313|12529408|12944480|11839803|11739748|12101227|11486039|10688665|10454591|10454552|10454584|9710627|9584162|9566894|9443911|11818069|10521653|11937641|12598317|9364045|10777774|11438577|11549719|11826111|9774968|9774967|10619024|11178871|11709154
rbl1->pml	9448006
rbl1->sp1	12788094|9694791|10913163|10022883|9566894|9819401|12730302|10436023|11433027|12954773|9148896
rela->creb1	10585438
rela->jun	10373501|7876165
rela->tp53	12887889
reln->bdnf	9728912

rin1->bcr	11027300
rin1->egf	11703925
ring1->rb	11583618
rpa1->tp53	10625690|9207066|11375889|10733572|8663296|10064605|10436023|10982866|10747950|11157767
rpl22->fn1	10217776
rrm2->vegf	12482957
rybp->yy1	11953439|12628927
sap18->hdac1	9150135
sap30->hdac1	12788099
scap->flj10261	12202038|12890500|12727870|12773382|12754279|12535518
scap1->fyn	12171928|11909961|9755858|9195899|9748251
sgk->bax	11842081
sh3glb1->bax	11259440
shb->crk	10878015
shb->ptpn11	12181353
shc1->egf	9710588|9694850|10330169|10613912|7559478|7544794|12048194|9346957|8702728|10395945|8702944|12897150|10567542
shc1->egfr	11016927|11382764|11133812|8636129|9642214|9685397|9813060|11375397|9710602|10888683|10514507|10400627|10567412|12496267|10781819|8789937|9442882|11084343|9384582|12101233|8662772|9851973|9710588|8895468|9877160|8702944|12743035|8626530|8824262|11723127|11907028|12897150|9488479|10395945|7541035|9268379|9857032|11134009|12297494|12414812|10882065|9765228|7535770|7608194|7622446|8626452|8798679|8631968|9368064|9733788|9430728|9478933|9371754|12588760
shc1->grb2	8939974|10085099|7559579
shp-1->bcr	11021804|11042209|9740800|11713294|9586639|10549631|11356834
shp-1->egfr	11042184|9733788|12887919|11157073|9287352|9305905|7673163|7545675|7559570|7531695|8647870|9038217|9507021|9418864
shp-1->p85	7537742|7744825|8626611|9083025|9110989|9162089|9188452|9188495|9195950|9211920|9452499|10488096|9050838|9171349|11337495
six2->aes	12441302
six4->aes	12441302
slc2a4->caveolin	11593015|12496259|10366592|9472045|9442106|12135605|11522683|11884617|11997522|11416153|11416134|10523618|9788240|10484611|11514613|11160826|11738086|9573373|12368209|11306717
smad1->smad4	10400705|10854429|10831835|12514223|12582250|9389648|12857866|9812998|11278251|11274206|11509558|12151385|12782279|9748228|11779505|10224145|10636910|11118211|9436979|9693372|9759503|10611968|9463378|9214507|10791953|10652350|11700304|10660046|12944489|10679014|11788714|12136093|12547711
smad2->sp1	11013220|10878024|11114293
smad3->sp1	11054406|10878024|11114293|11013220|10611754|11432852
smad4->sp1	10878024|11054406|11013220|10964912|11114293
smad4->vegf	10944227|12941698
smox->cycs	11181753
sos1->vws	10084996|9632564

sox10->sp1	11044731
sox2->sox10	12475944
sr->g72	9845327
srf->casp3	10777594|12874181
srf->creb1	9300412
srf->sp1	9295358
stat5->nr3c1	10713133|12552091|9989503|12391149|11287629|10454585|11160161|9915797|9813040|10601272|12754377
syk->bcr	12077122|8626447|12453414|9565626|12589045|11021804|9857187|7499277|8798459|7530715|8631803|8663063|8557712|9218456|10092671|10521450|10359809|12912915|11598012|10990449|11823472|10551821|8885868
taf4->creb1	11689682
taf4->sp1	11809795|11988536
tbk1->cdc42	10713183
tgfb2->cdk2	8798398
tmpo->vegf	9748518|12639965|12084458
tp53->bax	10851232|12480939|11852106|12574499|9826721|12667443|10753186|12943686|10545114|12084461|11834713|8702745|11489880|9315550|11050235|12045262|11815632|10702305|10506221|11278953|11350971|11904448|9122197|9649428|11782444|10207112|8939849|10570968|12433990|10436055|11961046|11395365|11932750|12971891|10559267|11893750|11756653|10205143|11179197|9525975|10027414|10980124|11804596|11827957|10531467|12091386|10869401|12091388|10688649|10930452|9182545|10980197|12799451|10487853|12459463|10744676|11864976|11943780|12538351|9445173|11940651|10925146|9242687|10574967|10747903|11335718|9177166|10688869|9054728|11080152|10329616|10805749|9891044|10710310|9436988|10514395|11395384|10526171|12062426|12586343|9006981|11438577|9660917|9774967|12379243|10867216|9852160|10446202|11054421|11875039|9454845|10479688|11477076|10037682|10982799|11836241|10547702|9707426|12438386|11997498|11731108|10518217
tp53->psen1	10942770|12556443|11904448|10805794|10446206
tp53->sp1	9380755|7649977|11384995|12665570|11546792|11842096|11278953|9685344|11350951|11375983|11279192|12208997|11844788|9169475|11427532|9275177|12771022|9885239|12805207|12842909|10364153|9774636|12034829|11844794
tr->ppara	9295326|9065436|7629123
tradd->fadd	8681376|9343360|12517920|12887920|10848577|9039262|8875942|8565075|9054379|10521444|10753878|10952991|11342564|11777919|11821416|12881424|9733516|9430229|9427646|10559258|11085984|11880365|12351681|11353862|10688869|8808629|11514590|9425165|11598011|10880445|9506948|11755217|9430227|10894161
tradd->krt14	11684708
trp-2->sox10	11156606
trrap->ppara	11931763
trrap->tp53	12660246
vcam1->igbp1	7534762|7499396|8663265|9507028|9677375|9348292|8621804|9012738|8798624|9539702|12354388|12626659
vcl->e-cadherin	9535896|9405455|10087272|9110993|9341178|9792660|10660044|10601344|9700171|9628899|11451997|11029037|10433943|10974003
x123->tp73	12519788
yy1->atf6	11595740|10866666
yy1->sp1	9819401|11955626|11514536|12788099|10514422

yy1->tp53	10787423

D.2.2 This table lists all of the arcs that exist on the graph. For each arc there is a list of the action types (in the Geneways 6.0 database) from the statements that were extracted from sentences in support of each arc (e.g. abl1 inhibits eif4e).

ARC	ACTION TYPES
abl1->eif4e	increase, inhibit
abl1->pxn	associate, coprecipitate, associate, contain, mediate, phosphorylate, required for
abl1->tp53	coexpress, degrade, depend on, elevate, enhance, form a complex, increase, induce, inhibit, interact, link, modulate, activate, promote, regulate, require, required for, stimulate, ubiquitinate, inactivate, lead to, affect, associate, bind
aes->creb1	inhibit
ahr->arnt	interact, affect, associate, phosphorylate, bind, signal, coimmunoprecipitate, colocalize, contain, form, activate, form a complex, add, independence, interact
ahr->egf	alter
akap5->g72	associate
ampk->cftr	interact, modulate, phosphorylate, regulate, result in, activate, associate, bind, coexpress, inhibit
ang->jun	activate, affect, induce, bind, regulate, result in, stimulate
ap-2->egfr	interact, bind, required for, associate, require, associate, coimmunoprecipitate
ap3->centg2	modify
ap3->hspg2	affect
apaf1->bcl2l1	associate, bind, coimmunoprecipitate, colocalize, form a complex, interact, regulate
apbb1->app	alter, mediate, enhance, modulate, produce, regulate, suppress, affect, associate, bind, cause, connect, increase, interact, link
app->psen2	regulate, require, bind, coimmunoprecipitate, inhibit, associate
app->tp53	affect, regulate, activate
arnt->psen2	recruit
atf3->creb1	bind
atf3->tp53	bind, depend on, down-regulate, require
atf5->ngfb	depend on
atp8a2->blmh	bind
atr->chek1	link, mediate, phosphorylate, regulate, required for, mediate, abrogate, activate, include, influence, lead to
baat->ppara	modulate
bace1->app	associate, bond, cause, cleave, coexpress, cut, induce, mediate, release, elevate, generate, produce, colocalize, abrogate, increase, limit, modify, truncate, affect, regulate, generate, produce
bad->bcl2l1	antagonize, dissociate from, down-regulate, form, inactivate, inhibit, interact, lead to, phosphorylate, required for, suppress, associate, bind, include, promote, antagonize, attenuate, bind, cause, coimmunoprecipitate, control, coprecipitate, dephosphorylate
bad->sos1	required for
bak1->bax	immunoprecipitate, link, modulate, overexpress, require, synergize, associate, bind, colocalize, depend on, form a complex,

	homolog
bard1->brca1	abolish, affect, associate, bind, block, coexpress, interact, colocalize, promote, depend on, regulate, down-regulate, require, elevate, required for, enhance, stimulate, increase, induce, affect
bard1->tp53	induce, interact, required for, alter
bax->bcl2	alter, antagonize, associate, because of, bind, block, coimmunoprecipitate, contain, coprecipitate, depend on, form, down-regulate, interact, repress, form a complex, homolog, immunoprecipitate, include, induce, inhibit, interact, link, modulate, overexpress, promote, regulate, require, required for, stimulate, up-regulate, abolish, abrogate, account for, affect
bccip->cdk2	associate
bche->atp8a2	hydrolyse, require, activate, add, bind, catalyze, depend on, form
bche->e2	acetylate, regulate, activate, require, add, associate, bind, catalyze, coexpress, combine, conjugate, contain, depend on, enhance, form, inhibit, interact, precede, promote
bche->e3	activate, add, associate, contain, interact
bcl2->psen1	associate, interact
bcl2l1->bax	lead to, result in, suppress, interact, abolish, activate, affect, alter, antagonize, associate, attenuate, bind, block, cause, coexpress, contain, encode, enhance, form, hinder, homolog, affect, associate, coimmunoprecipitate, immunoprecipitate, liberate, immunoprecipitate, inactivate, include, inhibit, promote, regulate, release, require
bcl2l1->psen1	associate, influence
bcl2l1->psen2	associate, influence
blr1->lta	induce
bmp2->smad1	activate, induce, phosphorylate, regulate, signal, stimulate, stimulate, affect, elicit, mediate, result in
bmp4->vegf	synergize
bnip3->bax	interact, substitute
bnip3->f2	affect, induce, lead to, promote
brca1->sp1	associate, bind, contain, interact, repress
brf1->mapk1	bind
brf1->tp53	associate
calm1->camk2g	activate, affect, associate, bind, induce, influence, inhibit, depend on, modulate, phosphorylate, promote, result in, stimulate, include, regulate
calm1->rgs4	bind
camk2g->nsf	phosphorylate
cap1->calm1	bind, encode, interact
cas->pxn	associate, bind, block, coprecipitate
cas1->il18	activate, required for
casp3->psen2	generate, cleave, inactivate
casp8->psen1	cleave
casp8->psen2	cleave
cav1->app	abrogate, associate, bind, form a complex, interact, link, regulate
cav3->app	alter, bind, colocalize, form a complex, immunoprecipitate, interact, promote
caveolin->apoer2	immunoprecipitate
cbl->akt1	lead to

cbl->bcr	associate, phosphorylate
cbl->egfr	accelerate, affect, associate, bind, coimmunoprecipitate, down-regulate, enhance, interact, depend on, link, mediate, increase, mediate, induce, recruit, required for, induce, inhibit, colocalize, stimulate, contain, promote, regulate, form, suppress, ubiquitinate
cblc->syk	interact
ccl4->sos1	require, induce
ccnd1->tr	activate, associate, interact, mediate, repress, transactivate
ccne2->cdk2	associate, bind
ccs->il10	induce
ccs->jun	induce, induce
cd160->sos1	affect, inhibit
cd28->pp2a	associate, bind, coprecipitate
cdk2->sp1	interact, phosphorylate
cdk2ap1->cdk2	result in, suppress, associate, coprecipitate, interact, regulate
cdk5->mapt	induce, mediate, modify, phosphorylate, affect, associate, bind, promote, catalyze, result in, cause, enhance, hyperphosphorylate, include, lead to
cdkn2a->mdm2	depend on, inactivate, stimulate, abrogate, affect, antagonize, associate, bind, block, bind, cause, coexpress, colocalize, contain, coprecipitate, degrade, form a complex, increase, induce, inhibit, bind, inhibit, interact, lead to, bind, block, interact, cause, lead to, inhibit, limit, interact, mediate, modify, modulate, promote, regulate, require, result in
ced-4->bcl2l1	required for, associate, bind, coimmunoprecipitate, interact
cfdp1->eif4e	bind, interact
cftr->atp8a2	synthesize, require, result in, add, affect, alter, associate, bind, catalyze, control, couple, depend on, elevate, enhance, interact, mediate, modulate, regulate, release
cftr->vws	bind, promote, produce
chd3->hdac1	associate
chd3->hdac2	associate
chd4->hdac1	associate
chd4->hdac2	associate
chek1->g72	phosphorylate
chrd->bmp4	inhibit, repress, antagonize, immunoprecipitate, bind, inhibit, inactivate, mediate, antagonize, block, degrade, disrupt
cks1b->cdk2	associate, bind, block, interact
clathrin->ap-2	associate, bind, colocalize, depend on, needed for, recruit, assemble, contain, enhance, form, increase, interact, recruit, required for, assemble
clk1->eif4e	affect
cls->cftr	affect
cls->egf	stimulate
coh1->fn1	bind
creb1->jun	activate, inhibit, precede, bind, interact, because of, contain, express, induce, phosphorylate, regulate, result in
creb1->sp1	interact
crk->pxn	associate, bind, colocalize, couple, down-regulate, elevate, include, inhibit, interact, recruit

crk->vegf	synergize, depend on
crkl->bcr	associate, bind, coprecipitate
cul1->myc	associate, coimmunoprecipitate, colocalize
dag1->pkd	activate, bind, cause, induce, inhibit, interact, mediate, recruit, regulate, required for, stimulate
dapk3->daxx	associate, bind, coimmunoprecipitate, colocalize, form, mediate, recruit
daxx->tp53	acetylate, bind, inhibit, interact, mediate, repress
dcn->egfr	attenuate, bind, cause, induce, inhibit, interact, lead to, mediate, suppress, cause, down-regulate, activate, activate, affect, mediate, associate, regulate
dlg1->akap5	alter, associate, bind, interact, link, required for
dp->apc	couple, link
e-cadherin->psen1	bind, required for
e2->eralpha	trigger, inactivate, induce, mediate, modulate, activate, affect, associate, bind, cause, colocalize, combine, regulate, control, repress, depend on, require, down-regulate, stimulate, immunoprecipitate
e2f1->bax	cause, coexpress, increase
e2f1->sp1	recruit, associate, bind, mediate, synergize, required for
e2f1->sp1	recruit, associate, bind, mediate, synergize, required for
e2f4->ccnd1	bind, induce
e2f5->e2f1	bind, regulate
edf1->calm1	bind, interact, bind
egf->eif4e	enhance, activate
egf->jun	regulate, stimulate, affect, alter, activate, contain, induce, recruit, transactivate
egf->sp1	accelerate, affect, bind, generate, induce, interact, mediate, require, stimulate
egfr->mapk1	activate, mediate, affect, signal, block, cause, mediate, couple, signal, induce, inhibit, lead to, produce, phosphorylate, regulate, required for, result in, associate, stimulate, bind, mediate, transactivate
egfr->rgs4	phosphorylate
egln1->cycs	cause
egr1->psen2	bind, interact
egr1->tp53	bind, induce, link, mediate, regulate, required for, associate
eif4e->ccnd1	increase, regulate, activate, induce
eif4g2->eif4e	associate
emx2->wnt1	bind, regulate, repress
ep300->tp53	inactivate, increase, acetylate, induce, activate, influence, add, synergize, inhibit, affect, transactivate, interact, associate, ubiquitinate, link, mediate, modulate, produce, bind, catalyze, coexpress, connect, promote, control, react, degrade, recruit, dissociate from, regulate, encode, remove, enhance, repress, form, required for, result in, signal, stimulate
eps15->egf	depend on, homolog
eps15->egfr	inhibit, recruit, required for, coimmunoprecipitate, phosphorylate, associate, bind, colocalize
eralpha->xbp1	recruit
erbb3->nrg1	bind, respond to, mediate, depend on, affect
erbb3->rgs4	form a complex, immunoprecipitate, interact
ern1->atf6	activate, induce, phosphorylate, regulate, required for

exo1->ap3	lead to
fadd->casp8	result in, signal, activate, assemble, associate, bind, lead to, mediate, recruit, regulate, alter, coexpress, inhibit, interact, mediate, require, interact, enhance, add, mediate, cause, lead to, recruit, contain, form, induce
fas->casp8	activate, induce, recruit, require, result in, cause, link, mediate, stimulate, trigger, lead to, bind, associate, couple, lead to, regulate, cleave, depend on, signal, form
fes->bcr	affect, associate, induce, interact, lead to, link, mediate, phosphorylate
flj10261->nsf	bind
fn1->bcr	abolish, depend on, required for
foxj1->cftr	regulate
frs2->grb2	associate, bind, coimmunoprecipitate, lead to, recruit, contain, coprecipitate, form, form a complex, interact, phosphorylate, regulate
fut1->il10	express, produce, release, produce
fyn->mapt	bind, interact, associate
fyn->vws	regulate
gab1->egfr	associate, bind, couple, depend on, mediate, recruit, signal
gab2->bcr	coimmunoprecipitate, increase, interact, recruit, regulate, required for, associate, bind
gab2->egf	account for, inhibit
glur2->grm3	contain, homolog, include, associate, coexpress
glur2->nsf	associate, bind, coimmunoprecipitate, colocalize, encode, interact, require, required for
grap2->grb2	homolog, interact
grb2->bcr	bind, coimmunoprecipitate, form a complex
grin2b->camk2g	inhibit, interact, mediate, phosphorylate, affect, bind, coexpress, contain, depend on, immunoprecipitate, induce
grip1->glur2	bind, interact, recruit, coimmunoprecipitate, colocalize
grp->atf6	induce, overexpress
gsk-3->mapt	associate, catalyze, immunoprecipitate, needed for, induce, inhibit, phosphorylate, mediate, activate, affect, react, phosphorylate, result in, hyperphosphorylate, include, increase
gtf2i->atf6	associate, coimmunoprecipitate, interact
gtf2ird1->gtf2i	homolog, interact
hbp1->rb	associate, bind, block, contain, immunoprecipitate, interact, repress
hbp1->wnt1	inhibit
hck->bcr	interact
hd->sp1	inhibit, bind, coimmunoprecipitate, immunoprecipitate, interact, recruit
hdac1->tp53	inhibit, acetylate, associate, cause, coexpress, deacetylate, induce, interact, mediate, modulate, promote, regulate, result in
hdac2->sp1	associate, bind
hdac9->creb1	enhance
hdac9->pml	coimmunoprecipitate, inhibit, interact, bind
hdac9->sp1	associate, required for
hdac9->tr	associate, bind
hgs->egf	depend on, inhibit
hgs->sos1	depend on

hipk2->pml	colocalize, interact
hipk2->tp73	interact
hlf->creb1	bind, bind
hlf->gc	interact
hlf->il8	block, modify
hlf->vws	add, bind, inhibit, interact, promote
hps1->ap3	associate
hrmt1l2->yy1	bind, depend on
hspa5->atf6	associate, bind, dissociate from, regulate, repress, required for
hspa5->atp8a2	block, depend on, hydrolyse, add, bind, depend on, form, inhibit, interact, mediate, regulate, release, require
hspa8->atp8a2	stimulate, add, bind, catalyze, depend on, immunoprecipitate, increase, mediate, regulate, require
hspa8->cftr	associate, bind, form, interact
hspa8->tp53	associate, bind, form a complex, interact, lead to, required for
hspd1->casp3	promote, stimulate, activate, associate, immunoprecipitate, mediate
icos->nfatc1	regulate, enhance, regulate
igbp1->g72	associate
igf1->nrg1	combine
igfbp1->igf1	abolish, add, affect, bind, determine, enhance, inhibit, block, lead to, stimulate, modify, regulate
il18->tp53	bind
il18->vws	depend on, determine, require, synergize, control
il5->ccnd1	induce
irf->sp1	affect
irf->vws	stimulate
jun->ap3	activate, assemble, bind, regulate, required for, result in, associate, attach, recruit
jun->sp1	interact, bind, combine, homolog
jun->vws	activate, bind, combine, down-regulate, enhance, mediate, inhibit
kcna4->fyn	coprecipitate
kl->il10	combine, synergistic action
krt14->plat	inhibit
lef1->ccnd1	bind, enhance
lef1->creb1	bind
lrp->app	mediate, modify, modulate, regulate, affect, alter, associate, bind, degrade, form a complex, immunoprecipitate, include, influence, result in, interact, increase, link
lta->tp53	affect, form a complex, inactivate, inhibit, interact, lead to
lu->egf	inhibit, required for
maea->fn1	interact
mapk1->myc	block, mediate, phosphorylate, phosphorylate, required for, activate,
mapk8->mapk8ip1	based on, bind, phosphorylate, regulate, coimmunoprecipitate, colocalize, dissociate from, immunoprecipitate, interact, mediate, recruit, result in, associate
mapk8->myc	associate, bind, lead to, phosphorylate, associate, form

mapk8ip1->apoer2	bind, coprecipitate
mapt->apoe	depend on, associate
mapt->psen1	associate, bind, colocalize
max->myc	require, required for, form, antagonize, inhibit, associate, interact, abrogate, bind, antagonize, associate, regulate, suppress, repress, block, coprecipitate
mdc1->brca1	result in, abolish, affect, interact, regulate, required for
mdm2->psen2	associate, recruit
mdm2->sp1	alter, associate, bind, immunoprecipitate, inhibit, interact, regulate, release
mdm2->tp53	repress, due to, elevate, form, form a complex, independence, influence, abolish, abrogate, accelerate, acetylate, activate, add, affect, initiate, alter, limit, link, mutate, mediate, phosphorylate, antagonize, arrest, as a result of, associate, attenuate, bind, block, carry, catalyze, cause, coexpress, coimmunoprecipitate, colocalize, control, contain, coprecipitate, degrade, depend on, determine, disrupt, down-regulate, enhance, immunoprecipitate, inactivate, increase, induce, inhibit, interact, lead to, mediate, modify, modulate, precede, promote, recruit, regulate, release, repress, require, required for, restrain, result from, result in, stimulate, synthesize, transactivate, trigger, ubiquitinate, up-regulate
mef2d->egf	inhibit
mitf->bcl2	modulate, bind, link, modulate, regulate
mitf->tyrp1	control, regulate, bind, regulate
miz1->ep300	recruit
miz1->myc	bind, inhibit, suppress, arrest, associate, form, increase, interact, mediate, regulate
mkl1->srf	activate, associate, bind, induce, inhibit, interact, coimmunoprecipitate, form a complex, form, required for, signal
mlana->egf	stimulate
mllt4->bcr	bind, colocalize, connect, coprecipitate, depend on
msx2->bmp4	depend on, inhibit, precede
myc->bcl2	alter, down-regulate, induce, inhibit, mediate, suppress, synergize, modify, overexpress
myc->pkd	associate
myc->sp1	interact, associate, bind, disrupt, inhibit, interact, require
myc->tp53	mediate, modulate, synergize, enhance, increase, interact, lead to, link, promote, regulate, result in, activate, antagonize, inactivate, induce, inhibit, abrogate, sensitize, add, signal, alter, associate, cause, depend on, disrupt
nab1->egr1	regulate, link, bind, inhibit, interact, repress, require
nab2->egr1	interact, modulate, regulate, repress, inhibit, result in
napa->nsf	activate, add, bind, enhance, homolog, inhibit, interact, mediate, regulate, stimulate
ncoa1->ccnd1	required for
ncoa2->tr	activate, enhance, affect, enhance, interact
nedd8->cul2	conjugate, modify, conjugate, conjugate
nedd8->pml	conjugate, modify
nes->tp53	block, degrade, inhibit, regulate
nes->xpo1	regulate, associate, required for, bind, block, depend on, form a complex, mediate, enhance, form a complex, interact
nf-kappab->sp1	combine, add, affect, assemble, bind, dissociate, interact, recruit, synergize, synergy, activate
nfatc1->vegf	mediate, induce, required for
ngfb->sp1	activate, alter, induce, interact

nos3->calm1	interact, add, associate, bind, depend on, form a complex, immunoprecipitate, independence, inhibit
nos3->caveolin	alter, associate, bind, block, coimmunoprecipitate, colocalize, contain, dissociate from, immunoprecipitate, include, interact, phosphorylate, regulate
notch2->jun	inhibit
nov->egf	bind, contain, overexpress
nox1->ang	required for, up-regulate
nox1->vegf	couple, induce, mediate, regulate
nrf1->cycs	mediate
nrf1->sp1	interact, synergize
nrg1->egf	inhibit, block, result in, induce, release, bind, contain, inhibit
nsf->atp8a2	add, bind, catalyze, cleave, contain, depend on, disrupt, induce, mediate, require
ogt->ap3	affect
ogt->sp1	modify, affect, inhibit, regulate, repress
ogt->tp53	link, modify
p120->e-cadherin	coimmunoprecipitate, colocalize, coprecipitate, due to, increase, interact, associate, regulate, bind, required for, block
p62->tp53	coprecipitate
p85->cdc42	associate, bind, enhance, interact, mediate
p85->egfr	associate, bind, recruit, associate
p85->erbb3	associate, bind, immunoprecipitate, recruit
parp1->yy1	bind, interact
pax2->egf	increase
pax2->tp53	inhibit, repress
pc->prb2	add, associate, combine, contain, form a complex
pdzk1->cftr	interact, modulate, regulate, associate, enhance, immunoprecipitate, coimmunoprecipitate, colocalize
pkd->syn2	phosphorylate
plat->sp1	down-regulate, increase, induce, lead to, result in
plod1->sos1	stimulate
plod1->vegf	increase, induce
plscr1->abl1	associate
pml->eif4e	recruit, required for, alter, antagonize, result in, assemble, associate, bind, colocalize, combine, depend on, inhibit, interact, modify, modulate, phosphorylate, regulate, repress, require, suppress
pp1->ppp1r15a	associate, bind, interact
pp2a->bad	dephosphorylate, initiate, interact, regulate, associate, bind, catalyze
pp2a->mapt	increase, dephosphorylate, hyperphosphorylate, lead to, phosphorylate, account for, induce, associate, interact, bind, may be responsible for, block, mediate, catalyze, cause, regulate, control, result in
ppara->creb1	bind
ppp1r15a->g72	recruit
prdx3->cycs	block, inhibit
prkcabp->glur2	alter, form, assemble, interact, induce, associate, bind, required for, coexpress, coimmunoprecipitate, colocalize, combine, contain

psen1->grm3	associate
psen2->psen1	react, form a complex, bind, coimmunoprecipitate, down-regulate, homolog, immunoprecipitate, lead to, modify
ptk2->egfr	associate, coimmunoprecipitate, colocalize, coprecipitate
ptpn11->egfr	bind, dephosphorylate, coprecipitate, enhance, associate
ptpn11->shc1	activate, affect, associate, coimmunoprecipitate, phosphorylate, recruit, assemble
pxn->igbp1	abolish, associate, bind, form a complex, inhibit, interact
pygm->apc	precede
qrsl1->vcam1	bind, down-regulate, mediate, up-regulate
rab38->tyrp1	required for
rab3a->apc	associate
rab3a->bdnf	inhibit, respond to, up-regulate
rab3a->calm1	associate, bind, increase, interact
rad9a->bcl2l1	associate, bind
rai->p85	bind, form a complex, recruit
ranbp2->cfdp1	bind, inhibit
rapsn->fyn	activate, associate, coexpress, coprecipitate, form a complex, increase, regulate
rb->mapk8	inhibit, interact, repress, suppress, activate, affect, associate, bind, cause, coimmunoprecipitate, enhance
rb->tp53	block, catalyze, coexpress, coimmunoprecipitate, depend on, form a complex, inactivate, induce, inhibit, interact, lead to, phosphorylate, regulate, trigger, activate, affect, bind
rbl1->pml	associate
rbl1->sp1	associate, bind, inhibit, interact, repress
rela->creb1	bind
rela->jun	increase, interact
rela->tp53	require, required for
reln->bdnf	depend on
rin1->bcr	interact
rin1->egf	inhibit, stimulate
ring1->rb	associate
rpa1->tp53	associate, bind, coimmunoprecipitate, coprecipitate, inhibit, interact, release
rpl22->fn1	bind
rrm2->vegf	required for
rybp->yy1	interact, interact
sap18->hdac1	interact, substitute, associate
sap30->hdac1	interact
scap->flj10261	bind
scap1->fyn	associate, bind, interact, promote, require
sgk->bax	affect
sh3glb1->bax	affect, associate, bind, colocalize, form, interact, promote, regulate
shb->crk	bind, interact
shb->ptpn11	associate, enhance, regulate

shc1->egf	associate, bind, block, coprecipitate, depend on, inhibit, interact, lead to, phosphorylate, recruit, required for, suppress
shc1->egfr	bind, interact, overexpress, phosphorylate, recruit, regulate, require, associate, activate, coprecipitate, couple, form, immunoprecipitate
shc1->grb2	associate, bind, phosphorylate
shp-1->bcr	activate, associate, mediate, recruit, regulate, signal
shp-1->egfr	associate, bind, interact, modulate, signal, suppress, cause, phosphorylate
shp-1->p85	associate, bind, dephosphorylate, induce, interact, mediate, modulate
six2->aes	interact
six4->aes	interact
slc2a4->caveolin	contain, associate, block
smad1->smad4	coexpress, phosphorylate, contain, require, depend on, form, enhance, induce, assemble, interact, associate, lead to, overexpress, bind
smad2->sp1	add, associate, enhance, interact, regulate
smad3->sp1	increase, interact, require, associate, bind, coimmunoprecipitate, enhance
smad4->sp1	associate, bind, coimmunoprecipitate, increase, interact
smad4->vegf	control, regulate, mediate
smox->cycs	affect
sos1->vws	activate, bind, mediate, respond to
sox10->sp1	interact, synergize
sox2->sox10	interact
sr->g72	associate
srf->casp3	activate, associate
srf->creb1	phosphorylate
srf->sp1	activate
stat5->nr3c1	associate, interact, required for, synergize, bind, coimmunoprecipitate, combine, depend on, require, interact, transactivate
syk->bcr	link, phosphorylate, recruit, activate, require, associate, required for, bind, signal, couple, trigger, depend on, interact
taf4->creb1	interact
taf4->sp1	associate, depend on
tbk1->cdc42	depend on
tgfb2->cdk2	inhibit
tmpo->vegf	add, regulate, due to
tp53->bax	abolish, activate, alter, associate, attenuate, bind, block, cause, colocalize, control, depend on, enhance, include, increase, induce, inhibit, interact, lead to, mediate, overexpress, phosphorylate, precede, promote, regulate, require, required for, result in, synergize, transactivate, up-regulate
tp53->psen1	bind, down-regulate, induce, inhibit, regulate, repress, suppress
tp53->sp1	affect, associate, bind, coimmunoprecipitate, connect, form a complex, include, inhibit, interact, mediate, modulate, require, synergize
tr->ppara	inhibit, required for, bind
tradd->fadd	bind, associate, enhance, interact, mediate, recruit, regulate
tradd->krt14	bind

trp-2->sox10	depend on
trrap->ppara	enhance
trrap->tp53	stimulate
vcam1->igbp1	bind, account for, interact
vcl->e-cadherin	couple, interact, recruit, associate, bind, coimmunoprecipitate, colocalize
x123->tp73	interact
yy1->atf6	interact, enhance, increase, interact
yy1->sp1	act synergistically, bind, repress, synergize
yy1->tp53	influence

D.3 List of Arc Types

This table list each of the action types which were identified by Geneways.

The first column identifies how we considered the verb.

- '+' were calculated towards activate arc.
- '-' were calculated towards inhibit arc.
- 's' were split evenly between the three possible values.
- '0' were calculated towards no effect.

The information from this table was used to calculate the arc prior-probability values as described in the math box.

s: split<br>+: activate<br>-: inhibit<br>0: no effect	Action Type<br>(actual connection word from the sentences).	Classification<br>L – Logical<br>P – Physical<br>O - Other
-	abolish	L
s	abrogate	L
s	accelerate	L
s	account for	O
s	acetylate	O
s	act synergistically	P
+	activate	L
s	add	O
s	add up	O
s	affect	L
s	aggregate	O
s	alter	O
s	analog	O
-	antagonize	L
s	arrest	L
s	as a result of	O
s	assemble	O
s	associate	O
s	attach	P
-	attenuate	L
s	attributable to	O
s	attributed to	O
s	based on	O
0	because of	L
s	bind	P
-	block	L
s	bond	O
s	break	O
s	carbamylate	P
s	carry	O
s	catalyze	P
s	cause	L
s	cleave	P
s	coexpress	O
s	coimmunoprecipitate	P
s	colocalize	P
s	combine	O
s	conjugate	O
s	connect	O
s	constrain	L
s	contain	O
s	control	L
s	coprecipitate	P
s	copurify	O
s	couple	O
s	cut	O
s	deacetylate	P
-	deactivate	L
s	deaminate	P
-	degrade	P
s	demethylate	P
s	depend on	L
s	dephosphorylate	P
s	depolymerize	P

s	determine	L		s	influence	L
s	digest	P		-	inhibit	L
s	diphosphorylate	P		+	initiate	L
s	disassemble	P		s	instigate	L
s	discharge	O		s	interact	L
s	disengage	O		s	iodinate	P
-	disrupt	L		s	join	O
s	dissociate	O		s	lead to	L
s	dissociate from	O		s	liberate	O
s	divide	O		-	limit	L
-	down-regulate	L		s	link	O
s	due to	O		s	mannosylate	P
s	elevate	L		s	may be responsible for	O
s	elicit	L		s	mediate	L
s	encode	O		s	mediate a signal	L
+	enhance	L		s	methylate	P
s	express	O		s	modify	O
s	form	O		s	modulate	L
s	form a complex	P		s	mutate	O
s	function synergistically	P		s	myristoylate	P
0	further	O		s	n-acetylate	P
s	generate	L		s	n-acylate	P
s	hasten	L		s	n-glycosylate	P
s	have an active role in	O		s	needed for	O
s	hew	O		s	o-glycosylate	P
-	hinder	L		0	ortholog	O
0	homolog	O		s	overexpress	O
s	hydrolyse	P		s	pair	O
s	hyperacetylate	P		s	paralog	O
s	hypermethylate	P		s	phosphorylate	P
s	hyperphosphorylate	P		s	photoreactivate	L
s	hypophosphorylate	P		s	polymerize	O
s	immunoprecipitate	P		0	precede	O
-	inactivate	L		s	prenylate	P
+	incite	L		s	produce	O
0	include	O		+	promote	L
+	increase	L		s	prompt	L
s	independence	O		s	proteolyze	P
+	induce	L		s	react	L

s	recruit	O	-	suppress activity of	L
s	regulate	L	s	synergistic action	L
s	release	O	s	synergistic association	P
-	remove	O	s	synergistic cooperation	P
s	replace	O	s	synergistic integration	L
-	repress	L	s	synergistic interaction	P
s	require	O	s	synergize	P
s	required for	O	s	synergy	P
s	respond to	O	s	synthesize	O
-	restrain	L	s	tie	O
s	result from	O	+	transactivate	L
s	result in	O	s	transcribe	O
s	secrete	O	s	translate	O
s	sensitize	L	+	trigger	L
s	separate	O	s	truncate	P
s	set free	O	-	ubiquitinate	P
s	sever	P	-	ubiquitinize	P
s	signal	L	s	unpair	O
s	splice	O	+	up-regulate	L
s	split	P	s	urge	L
+	stimulate	L	s	work synergistically	P
s	substitute	O			
-	suppress	L			

D.4. List of Brain Tissue Names

D.4.1 This is a list of the tissue names that we associated as being brain tissue names. The second columns gives the count of the number of PubMed abstracts which mention the tissue.

**TISSUE NAME**	# PUBMED ABSTRACTS
anterior commisure	11
anterior pituitary	10851
astrocytes	20788
astroglia	1315
brain	363447
brain astrocytes	325
brain capillary	1238
brain cortex	2540
brain hippocampus	119
brain stem	16503
brain striatum	306
brainstem	19493
buccal ganglia	230
buccal ganglion	166
buccal muscle	73
bursa of fabricius	1086
calvaria	2416
caudate	11800
central ganglia	123
central ganglion	35
cerebellar purkinje cells	1463
cerebellar purkinje neurons	371
cerebellum	25371
cerebral cortex	26672
cerebral ganglia	290
cerebral ganglion	310
cerebral pedal	29
cerebrospinal fluid	40483
cerebrum cortex	12
choroid plexus	4901
chromaffin granules	1281
circumoesophageal ganglion	1
cns	47955
coeliac ganglia	58
coeliac ganglion	106
coelomic fluid	256
colostrum	3789
conceptus membrane	6
corpora cardiaca	272
corpus callosum	5600
corpus striatum	3290
diencephalon	3148
dorsal root ganglia	3969
dorsal root ganglion	3664
embryonic brain	938
embryonic chondrocytes	27
embryonic spinal cord	312
ependymocyte	128
fetal brain	2832
fetal cerebellum	45
forebrain	14230
fornix	2355
frontal cortex	8201
frontal lobe	6797
ganglia	28260
ganglion	35538
glial	26627
glial cell	13098
glioblastoma	7401
glioma cell	5270
habenula	974
hindbrain	2290
hippocampus	38695
hypothalamic	40355
hypothalamus	34369
locus coeruleus	4805
medulla oblongata	3681

mesencephalon	2862
midbrain/hindbrain	267
myelencephalon	105
neonatal brain	863
neonatal brainstem	16
nerve	205548
nerve cord	1068
nerve endings	6334
neural arch cartilage	1
neural ganglion	31
neural plate	871
neural tube	6589
neuroglia	735
neuroglial cell	320
neuroglioma	42
neuron	225560
neurospongioma	2
neurula	607
occipital cortex	2002
occipital lobe	1945
parietal lobe	1979

pars intercerebralis	147
pons	5804
posterior tectum	17
purkinje cells	6914
purkinje neurons	866
schwann cell	6672
septum pellucidum	536
subcommissural organ	481
substantia nigra	10287
sympathetic ganglia	2443
sympathetic ganglion	1466
temporal cortex	2651
temporal lobe	11059
thalamus	15802
visceral ganglia	58
visceral ganglion	91
visceral mass	60
white-root	11
**106 TISSUES**	**910,221 ABSTRACTS**

D.4.2 This is a cross-list between the nodes and the brain tissue names which appeared in the same abstract. We used this co-occurrence of terms within the abstract to approximate the priors for the particular node being present in the brain.

NODE	BRAIN TISSUE(S)
abl1	nerve, neuron, cns, brain
aes	brain
ahr	ganglia, brain
akap5	neuron, brain, hippocampus, cns, neonatal brain, cerebral cortex, forebrain
akt1	neuron, cerebellum, brain, astrocytes, glial
ampk	brain, astrocytes, hypothalamic, neuron, hypothalamus, nerve
ang	
ap-2	neural tube, brain
ap3	cerebellum, purkinje cells, cerebellar purkinje cells, cerebrospinal fluid, cns, neuron, nerve, brain, fetal brain
apaf1	neuron, hippocampus, brain, embryonic brain
apbb1	brain, neuron, nerve, fetal brain
apc	neuron, brain, fetal brain, cerebellum
apoe	hippocampus, cerebellum, temporal cortex, brain, frontal cortex, neuron, cns, pons, nerve, astrocytes, glial cell, glial, cerebrospinal fluid, glioblastoma, temporal lobe, occipital cortex, frontal lobe
apoer2	hippocampus, purkinje cells, brain, cerebral cortex, neuron, embryonic brain, nerve, choroid plexus
app	neuron, brain, cerebrospinal fluid, astrocytes, nerve, hippocampus, forebrain, corpus callosum, glial, glioma cell, temporal lobe, cerebral cortex, cns, frontal cortex, neuroglioma, hypothalamic, brain capillary, choroid plexus, glioblastoma, fetal brain
arnt	
atf3	nerve, neuron, brain
atf5	brain, neuron
atf6	
atp8a2	cns
atr	
baat	
bace1	brain, brain cortex, neuron, temporal cortex, frontal cortex, neural tube, hippocampus, astrocytes, cerebellum, pons
bad	neuron, nerve
bak1	brain, frontal lobe, nerve, neuron
bard1	
bax	brain, glioblastoma, glial, nerve, neuron, substantia nigra
bccip	brain
bche	brain, neuroglia, cns, neuron, nerve, hypothalamus, thalamus, fetal brain, ganglia
bcl2	astrocytes, cns, neuron, brain, glioblastoma, glioma cell, cerebellum, frontal cortex, frontal lobe, ganglia, nerve, dorsal root ganglia
bcl2l1	brain, mesencephalon, neuron, astrocytes, cns, frontal lobe
bcr	
bdnf	nerve, hippocampus, brain, neuron, forebrain, astrocytes, glial cell, glial, occipital cortex, frontal cortex, cns, hypothalamus, cerebral

	cortex, caudate, temporal cortex
blmh	astrocytes, brain
blr1	astrocytes, cns
bmp2	neuron, nerve, schwann cell, glioma cell, brain, calvaria
bmp4	neuron, nerve, schwann cell, neural tube, calvaria
bnip3	
brca1	
brf1	
calm1	neuron, brain
camk2g	hippocampus, cerebral cortex, neuron, cns, brain
cap1	glioblastoma
cas	neuron, brain
cas1	brain
casp3	neuron, brain, cns, hippocampus, cerebrospinal fluid, glioma cell, neural tube
casp8	astrocytes, cns, neuron, cerebrospinal fluid, brain, cerebellum, glioma cell
cav1	neuron, astroglia, cerebral cortex, astrocytes, glial, brain, glioma cell, glial cell, nerve
cav3	neuron
caveolin	neuron, astroglia, cerebral cortex, astrocytes, glial, brain, glioma cell, fetal brain, nerve, glial cell
cbl	neuron
cblc	brain
ccl4	brain, cerebrospinal fluid, astrocytes, cns, glial, neuron
ccnd1	neuron, astrocytes, glioblastoma
ccne2	
ccs	brain, neuron, purkinje cells, astrocytes
cd160	
cd28	brain
cdc42	nerve, embryonic brain, neuron, brain, cns, glioblastoma
cdk2	brain, astrocytes, cns
cdk2ap1	
cdk5	neuron, brain, cns, hippocampus, caudate, thalamus, substantia nigra, corpus callosum, ganglia, brainstem, cerebral cortex, temporal lobe, nerve, astrocytes, ganglion, purkinje cells, cerebellar purkinje cells, dorsal root ganglia, nerve endings
cdkn2a	nerve, glioblastoma, astrocytes, cns, glioma cell, brain
ced-4	brain, fetal brain
centg2	hippocampus, cerebellum, brain, fetal brain, caudate, thalamus, corpus callosum
cfdp1	
cftr	brain, neuron, cns, hypothalamus, hypothalamic
chd3	brain
chd4	
chek1	
chrd	cerebellum

cks1b	
clathrin	brain, neuron, ganglia, glial cell, schwann cell, dorsal root ganglia, glial, nerve
clk1	brain, neuron, hypothalamus
cls	brain
coh1	brain
creb1	neuron, hippocampus, brain, astrocytes, temporal lobe
crk	nerve, neuron
crkl	nerve
cul1	brain
cul2	cns
cycs	neuron, hippocampus, brain
dag1	hippocampus, cerebellum, brain, cerebral cortex, neuron, nerve, schwann cell, purkinje cells, purkinje neurons, cerebellar purkinje neurons, astrocytes, glial
dapk3	
daxx	
dcn	glioma cell, cerebral cortex, neuron, brain
dlg1	neuron, cerebellum, astrocytes, hippocampus, glial cell, brain, purkinje neurons, cerebellar purkinje neurons
dp	
e-cadherin	schwann cell, brain, anterior pituitary
e2	glioblastoma
e2f1	brain, astrocytes, neuron, glial, cns
e2f4	neuron
e2f5	
e3	brain, hippocampus, temporal cortex
edf1	
egf	brain, forebrain, neuron, nerve
egfr	nerve, neuron, glioblastoma, glioma cell, glial, brain, forebrain, astrocytes, hippocampus, cerebellum, fetal brain, cns, brain stem, brain astrocytes
egln1	neuron, nerve
egr1	hypothalamus, anterior pituitary, brain, nerve, neuron, glioblastoma, hindbrain
eif4e	
eif4g2	
emx2	brain, cerebral cortex, thalamus
ep300	astrocytes, neural tube, nerve, ganglion, brain, neuron, dorsal root ganglion
eps15	nerve, glial cell, glial
eralpha	brain, hypothalamic, neuron, hypothalamus, thalamus, cerebrospinal fluid, choroid plexus, anterior pituitary
erbb3	brain, schwann cell, neuron, cerebellum
ern1	brain, neuron
exo1	
f2	glioma cell

fadd	astrocytes, cns, hippocampus, neuron, substantia nigra, nerve
fas	hippocampus, cerebellum, brain, ganglion, sympathetic ganglion, anterior pituitary, neuron, astrocytes, glial, glioma cell, glioblastoma, glial cell, fetal brain, cns, cerebrospinal fluid, nerve
fes	ganglion, dorsal root ganglion, neuron
flj10261	
fn1	brain, glioblastoma, glial
foxj1	brain, brain cortex, cerebrospinal fluid, choroid plexus
frs2	nerve, neuron, glial cell, glial, neural tube
fut1	
fyn	hippocampus, brain, neuron, astrocytes, glioma cell, cerebral cortex, hypothalamic, nerve
g72	brain
gab1	glial cell, glial, nerve, glioblastoma
gab2	brain, nerve
gc	
glur2	cns, temporal lobe, neuron, brain, purkinje cells, cerebellar purkinje cells, hippocampus, cerebrospinal fluid, glial, cerebellum, glial cell, thalamus, cerebral cortex, corpus callosum, frontal cortex, caudate
grap2	
grb2	nerve, brain, astrocytes, hippocampus, neuron, purkinje neurons, cerebellar purkinje neurons, glioblastoma, cerebellum
grin2b	hippocampus, brain, cns, neuron, glial, ganglia, forebrain, cerebellum, cerebral cortex, nerve, fetal brain, brainstem, thalamus
grip1	brain, neuron
grm3	glial cell, glial, neuron, glioma cell, glioblastoma, astrocytes, hippocampus, temporal lobe, cerebellum, brain, caudate, thalamus, cerebral cortex, corpus callosum
grp	brain, astrocytes, neuron, nerve, glioblastoma
gsk-3	neuron, brain, cerebral cortex, cns, frontal cortex, nerve, hypothalamus, hippocampus
gtf2i	brain
gtf2ird1	brain
hbp1	
hck	brain, hypothalamic, neuron
hd	brain, neuron, caudate, nerve, hypothalamus, glial, brainstem, brain striatum, cerebellum, cerebral cortex, ganglia
hdac1	
hdac2	
hdac9	neurula, neuron, brain
hgs	schwann cell, brain
hipk2	neuron
hlf	brain
hps1	
hrmt1l2	neuron
hspa5	hippocampus, forebrain, neuron, brain
hspa8	neuron
hspd1	nerve, hypothalamic, neuron

hspg2	
icos	
igbp1	brain
igf1	neuron, hypothalamus, hypothalamic, brain, glioblastoma, astrocytes, fetal brain, glial, nerve, hippocampus, glial cell, caudate, cerebral cortex
igfbp1	nerve
il10	brain, glial cell, astrocytes, glial, nerve, anterior pituitary
il18	brain, cns
il5	nerve, neuron
il8	brain, cns, cerebrospinal fluid, astrocytes, hippocampus, neuron, substantia nigra
irf	brain, neuron, nerve
jun	glioblastoma, astrocytes, neuron, hippocampus, brain, cns, glial cell, glial, calvaria, nerve
kcna4	brain, cns, forebrain, neuron
kl	brain
krt14	
lef1	
lrp	hippocampus, cns, neuron, brain, astrocytes, hindbrain, brain capillary, glioblastoma, glial cell, glial
lta	nerve, glial cell, glial, astrocytes, cns
lu	
maea	
mapk1	brain, cerebrospinal fluid, astrocytes, glial, neuron, cerebral cortex, nerve, substantia nigra, glial cell, cerebellum
mapk8	neuron, brain, nerve, astrocytes, glial
mapk8ip1	brain, nerve, neuron
mapt	neuron, brain, cerebrospinal fluid, hippocampus, frontal lobe, glial cell, glial, nerve, nerve cord, corpus striatum, astrocytes, temporal lobe, frontal cortex, cerebellum, cerebral cortex, cns, substantia nigra, ganglia
max	
mdc1	
mdm2	glioblastoma, brain, astrocytes
mef2d	neuron
mitf	
miz1	
mkl1	brain, fetal brain
mlana	
mllt4	
msx2	brain, nerve
myc	neuron, cns, brain, glioblastoma
nab1	nerve, hindbrain
nab2	nerve, hindbrain
napa	brain, neuron
ncoa1	neuron, nerve

ncoa2	hypothalamic, brain
nedd8	neuron, ganglia, brain
nes	brain, glial cell, fetal brain, cns, glial, neuron, nerve, schwann cell, astrocytes, hippocampus, brain astrocytes, astroglia, ganglion, cerebellum, purkinje cells, neural tube, corpus callosum
nf-kappab	astrocytes, glial, hypothalamic, glioblastoma, brain, neuron, anterior pituitary, cns, hippocampus, nerve, brain capillary, glial cell, substantia nigra
nfatc1	
ngfb	nerve, neuron, brain, glial cell, glial, neurula, cns, forebrain, anterior pituitary, ganglia, sympathetic ganglia, dorsal root ganglia
nos3	nerve, brain, neuron, ganglion, astrocytes
notch2	hippocampus, cerebellum, brain, purkinje cells, neuron
nov	cerebrospinal fluid, brain, glioblastoma
nox1	
nr3c1	brain, hypothalamic, hippocampus
nrf1	neuron, brain
nrg1	hippocampus, brain, astrocytes, neuron, glioma cell, cerebellum, brainstem, purkinje cells, cerebellar purkinje cells, substantia nigra, ganglia, dorsal root ganglia, schwann cell, glial
nsf	brain, neuron, hippocampus, nerve endings, nerve, anterior pituitary
ogt	brain, nerve, neuron
p120	neuron, brain
p62	glioma cell
p85	neuron, brain, cerebrospinal fluid, nerve
parp1	brain, astrocytes, glial, neuron, cerebellum, brainstem, glial cell, substantia nigra
pax2	nerve, cns, brain
pc	brain, astrocytes, neuron, brain stem
pdzk1	
pkd	astrocytes, neuron, brain, cns
plat	hippocampus, cns, neuron, brain, cerebrospinal fluid, thalamus, nerve, astrocytes, purkinje cells, cerebellar purkinje cells, purkinje neurons
plod1	
plscr1	hippocampus, neuron
pml	ganglia, brain, glial cell, sympathetic ganglia, glial, neuron, cns
pp1	neuron, hypothalamus, brain
pp2a	
ppara	neuron
ppp1r15a	
prb2	
prdx3	brain, neuron
prkcabp	brain, neuron, hippocampus, cns, nerve
psen1	neuron, cerebral cortex, brain, hippocampus, glial, glial cell, ganglia, nerve, frontal lobe, brain cortex, embryonic brain, forebrain
psen2	neuron, glial, brain, cerebral cortex, nerve, astrocytes, hippocampus, cerebellum

ptk2	brain, embryonic brain, neuron, glioblastoma, nerve
ptpn11	hypothalamus, hypothalamic, neuron, glioblastoma, brain, nerve, fetal brain
pxn	nerve
pygm	nerve, brain
qrsl1	
rab38	
rab3a	neuron, brain, nerve
rad9a	brain
rai	nerve, brain, neuron
ranbp2	ganglion, neuron, brain, fetal brain
rapsn	nerve, brain, neuron
rb	cns, hippocampus, frontal cortex, substantia nigra, neuron, brain, glioblastoma, fetal brain, astrocytes
rbl1	neuron
rela	astrocytes, glial, glioblastoma, brain, neuron, anterior pituitary, cns, hippocampus, glial cell, nerve
reln	brain, neuron, hippocampus, cerebellum, brainstem, cerebral cortex, glial, temporal lobe, cerebrospinal fluid, cns, temporal cortex
rgs4	glioma cell, cerebellum, frontal cortex, ganglia, temporal cortex, brain, caudate, thalamus
rin1	
ring1	
rpa1	
rpl22	brain
rrm2	
rybp	
sap18	
sap30	
scap	
scap1	neuron
sgk	brain, neuron, astrocytes, glial
sh3glb1	brain
shb	brain
shc1	nerve, neuron, brain, embryonic brain, fetal brain, ganglia, ganglion, hypothalamic, cerebellum, glial cell, astrocytes, glial
shp-1	neuron, nerve
six2	ganglion
six4	hypothalamic, neuron
slc2a4	neuron, brain
smad1	astrocytes, neuron, nerve
smad2	neuron, brain, glioblastoma
smad3	anterior pituitary, astrocytes, glial cell, glial, neuron, brain, glioblastoma
smad4	astrocytes, glial cell, glial, brain, glioblastoma
smox	

sos1	nerve, schwann cell, neuron
sox10	ganglia, glial, glial cell, brain, fetal brain
sox2	brain
sp1	brain, cns, glioblastoma, astrocytes, nerve, neuron
sr	brain, nerve, neuron, substantia nigra, glial cell, glioblastoma, astrocytes, glial, cerebral cortex, cns
srf	
stat5	ganglion, nerve, neuron
syk	neuron, brain
syn2	neuron, brain, nerve
taf4	neuron
tbk1	
tgfb2	glioma cell, brain, glioblastoma
tmpo	nerve
tp53	neuron, brain, glioblastoma, glioma cell, fetal brain, glial, astrocytes, glial cell, choroid plexus, occipital lobe, cns, cerebellum, frontal cortex, nerve
tp73	neuron, brain, fetal brain, hippocampus, brain hippocampus
tr	anterior pituitary, brain, fetal brain, nerve, neuron, cerebrospinal fluid, astrocytes, cns
tradd	nerve, hippocampus, neuron, brain
trp-2	glioma cell, brain, glial cell, glioblastoma, glial
trrap	occipital lobe
tyrp1	
vcam1	brain, astrocytes, cns, hippocampus, nerve
vcl	neuron, embryonic chondrocytes
vegf	brain, ganglion, astrocytes, glioblastoma, glioma cell, neuron, glial cell, glial, cerebrospinal fluid, anterior pituitary, ganglia, nerve, dorsal root ganglion
vws	
wnt1	cerebellum, brainstem, brain, brain stem, neural tube, mesencephalon
x123	
xbp1	
xpo1	astrocytes
yy1	glioblastoma
